# Supplementary material for: A New Family of Homoleptic Copper Complexes of Curcuminoids: Synthesis, Characterization and Biological Properties
Source: Molecules. 2019 Mar 5;24(5):910. doi: 10.3390/molecules24050910 (PMC6429335; doi:10.3390/molecules24050910)
Supplement: Supplementary file 1 [file molecules-24-00910-s001.pdf]

Supplementary Information

A New Family of Homoleptic Copper Complexes of Curcuminoids: Synthesis, Characterization and Biological Properties

William Meza-Morales, Juan C. Machado-Rodriguez, Yair Alvarez-Ricardo, Marco A. Obregón-Mendoza, Antonio Nieto-Camacho, Rubén. A. Toscano, Manuel Soriano-García, Julia Cassani and Raúl G. Enríquez

Correspondence: cassani@correo.xoc.uam.mx (J.C.); enriquezhabib@gmail.com (R.G.E.); Tel.: +52-5554837255 (J.C.); +52-5556224404 (R.G.E.); Fax: +52-5556224404 (R.G.E.)

Table of Contents

NUCLEAR MAGNETIC RESONANCE 3

INFRARED SPECTROSCOPY 29

MASS SPECTROMETRY 39

DRX SINGLE CRYSTAL 49

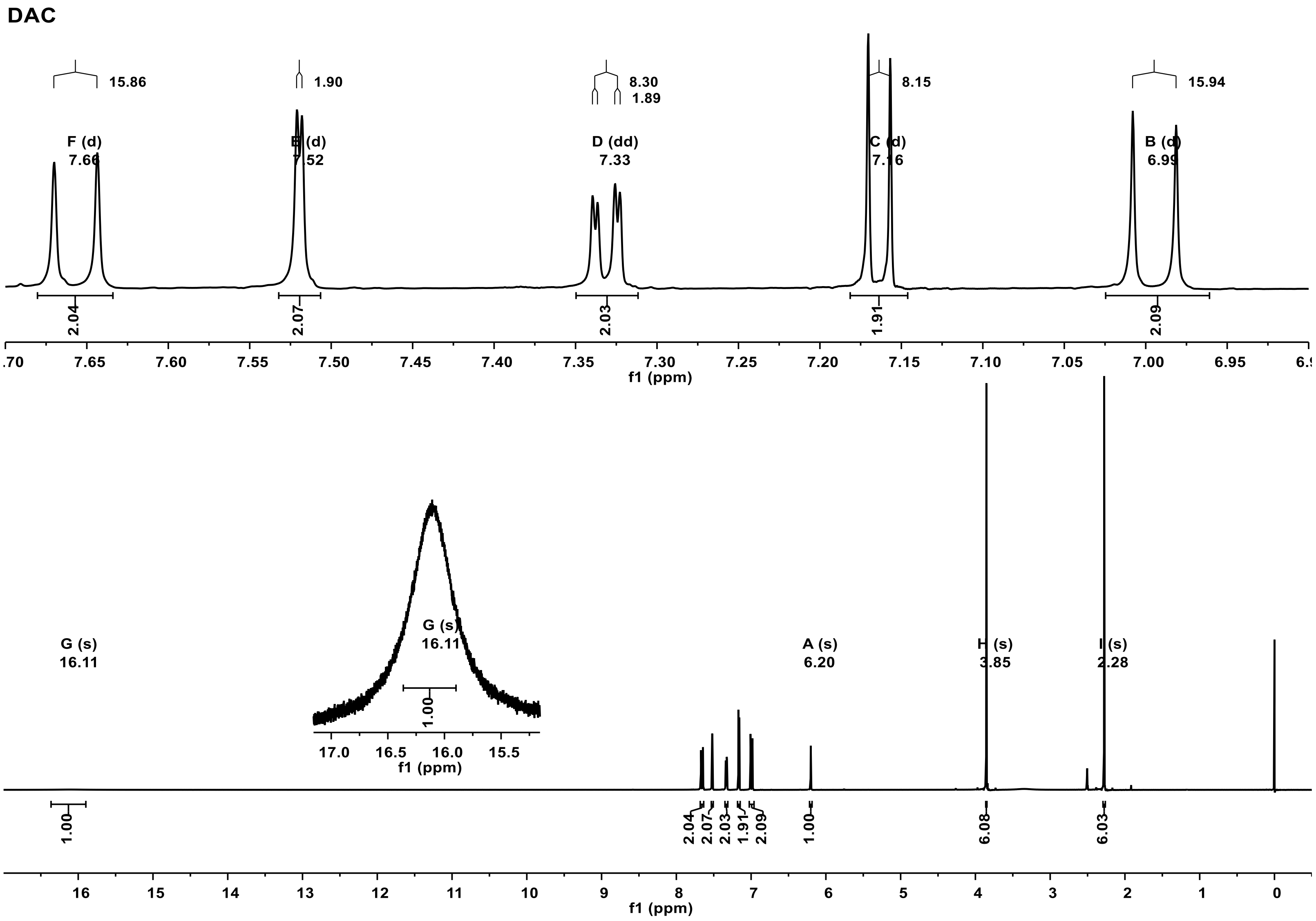

Figure S1. 500 MHz ^{1}H NMR spectrum of diacetyl-curcumin.

DAC

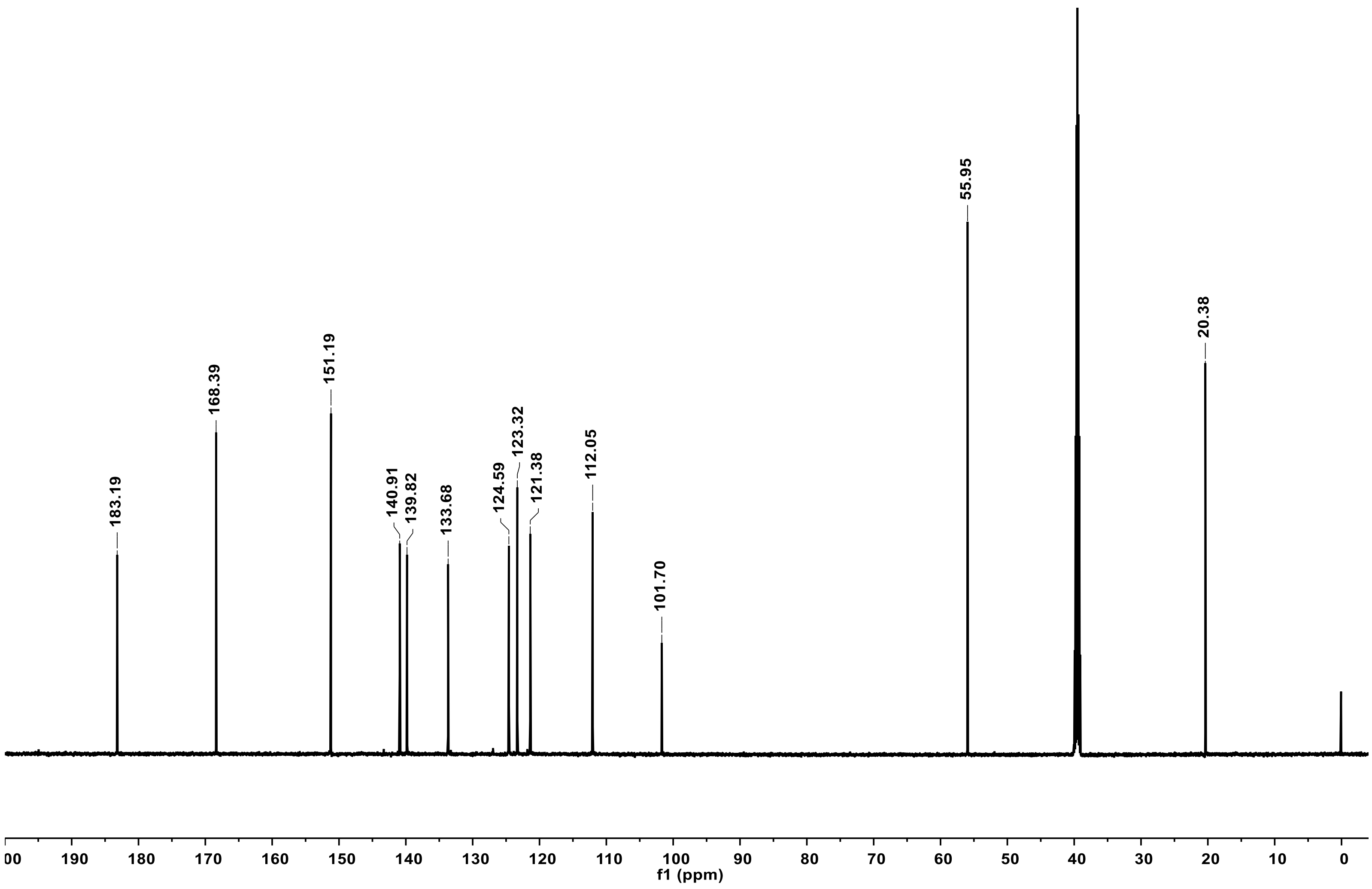

Figure S2. 125 MHz ^{13}C NMR spectrum of diacetyl-curcumin.

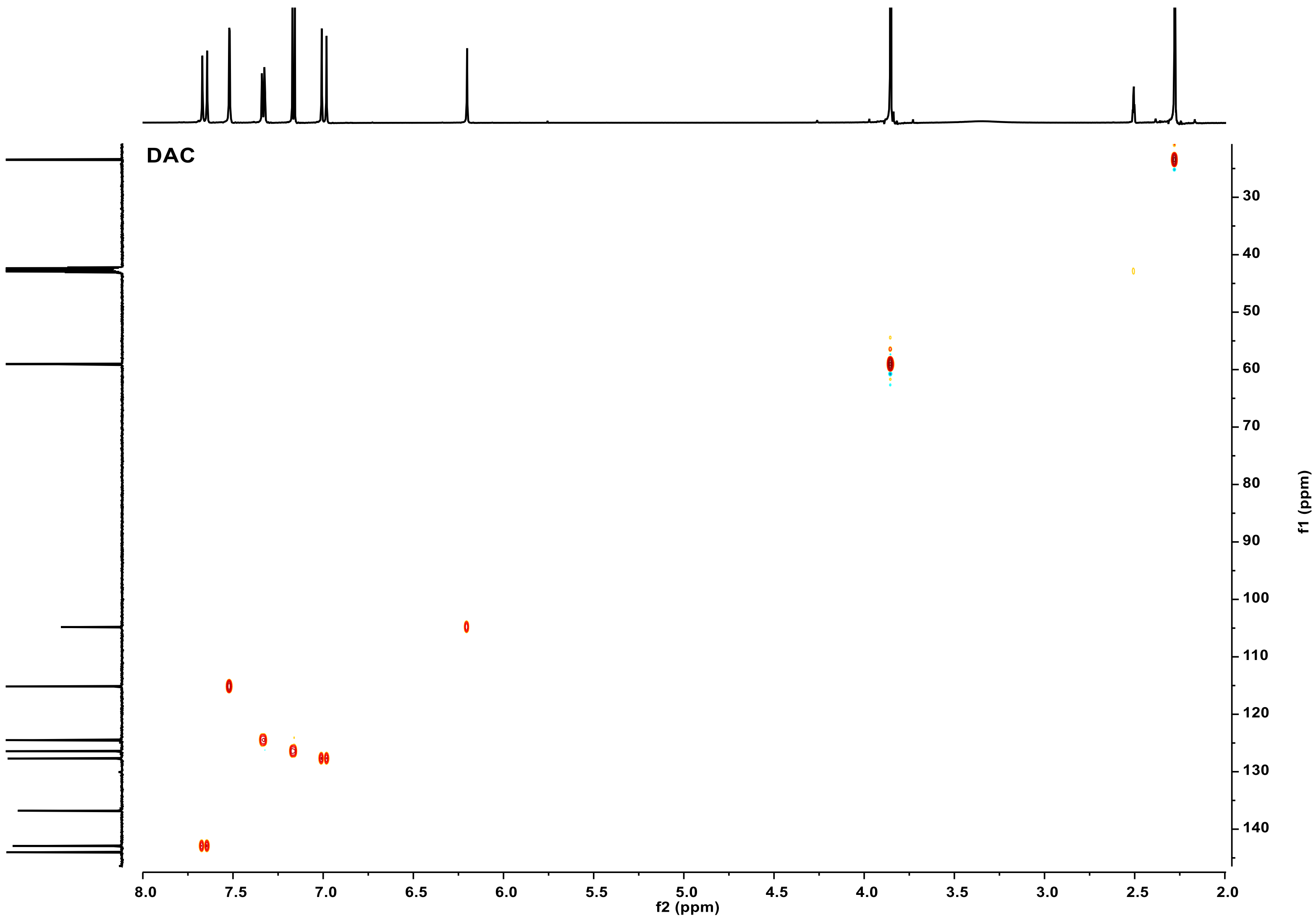

Figure S3. 500 MHz HSQC NMR spectrum of diacetyl-curcumin.

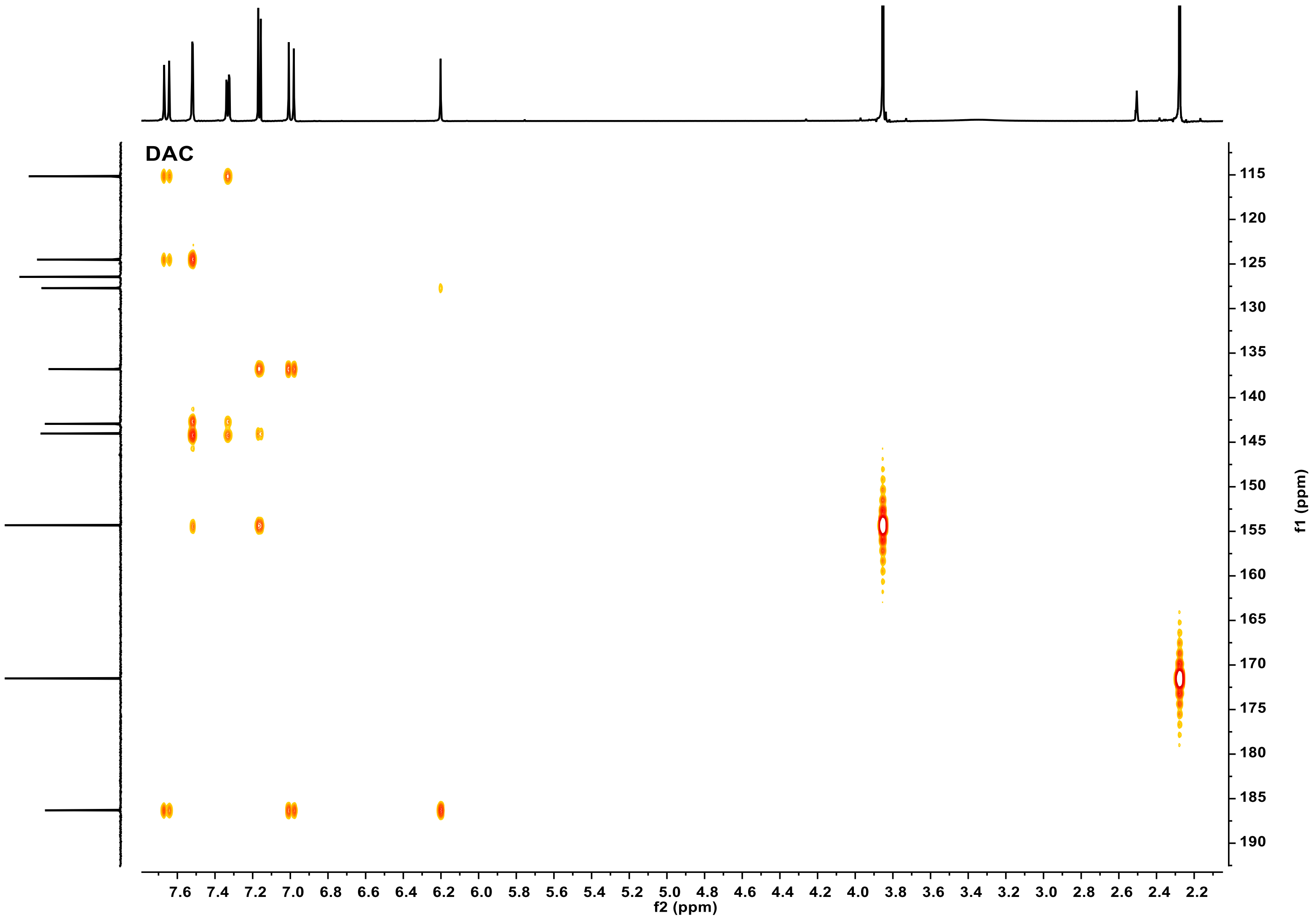

Figure S4. 500 MHz HMBC NMR spectrum of diacetyl-curcumin.

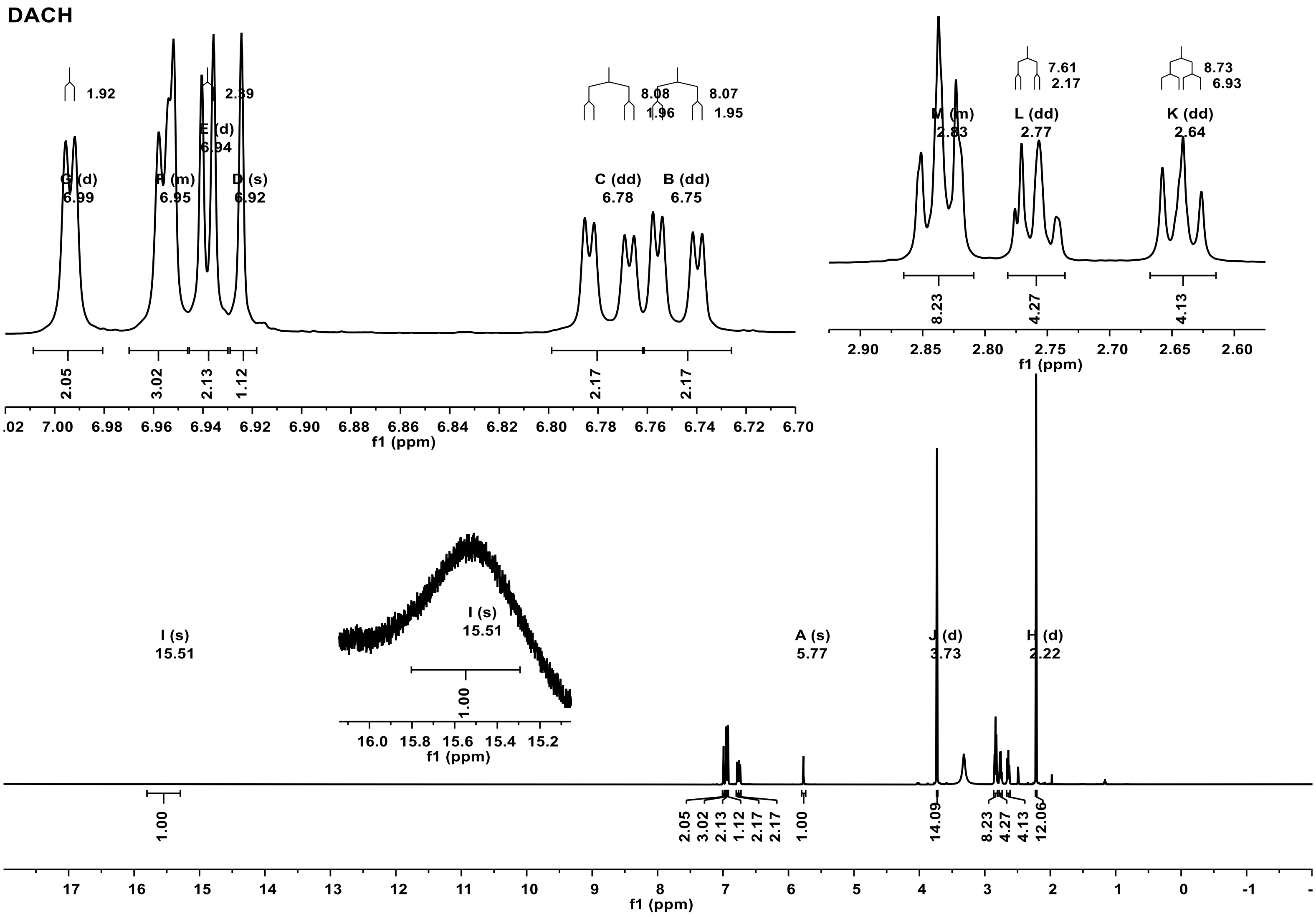

Figure S5. 500 MHz ^{1}H NMR spectrum of hydrogenated diacetyl-curcumin.

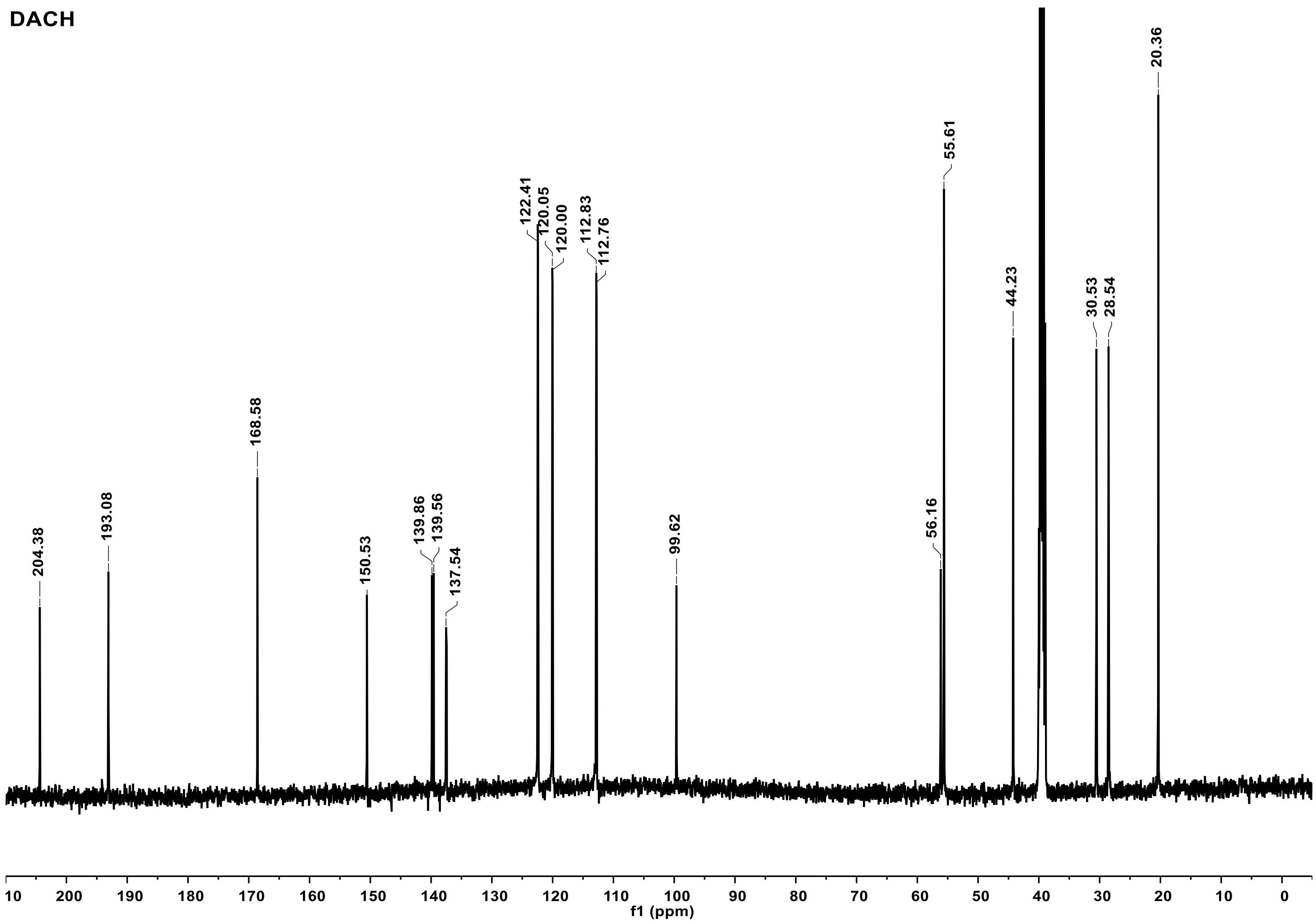

Figure S6. 125 MHz ^{13}C NMR spectrum of hydrogenated diacetyl-curcumin.

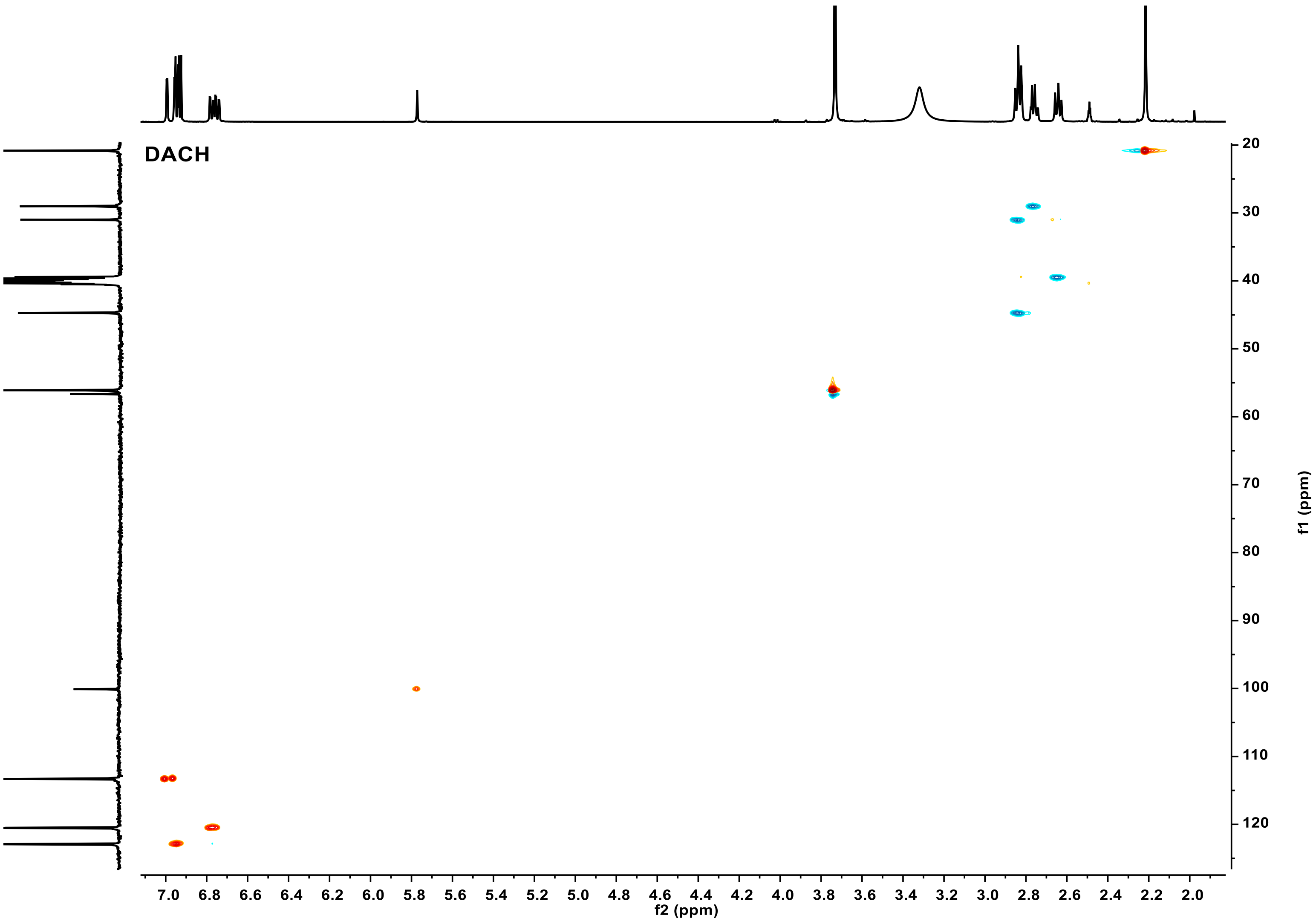

Figure S7. 500 MHz HSQC NMR spectrum of hydrogenated diacetyl-curcumin.

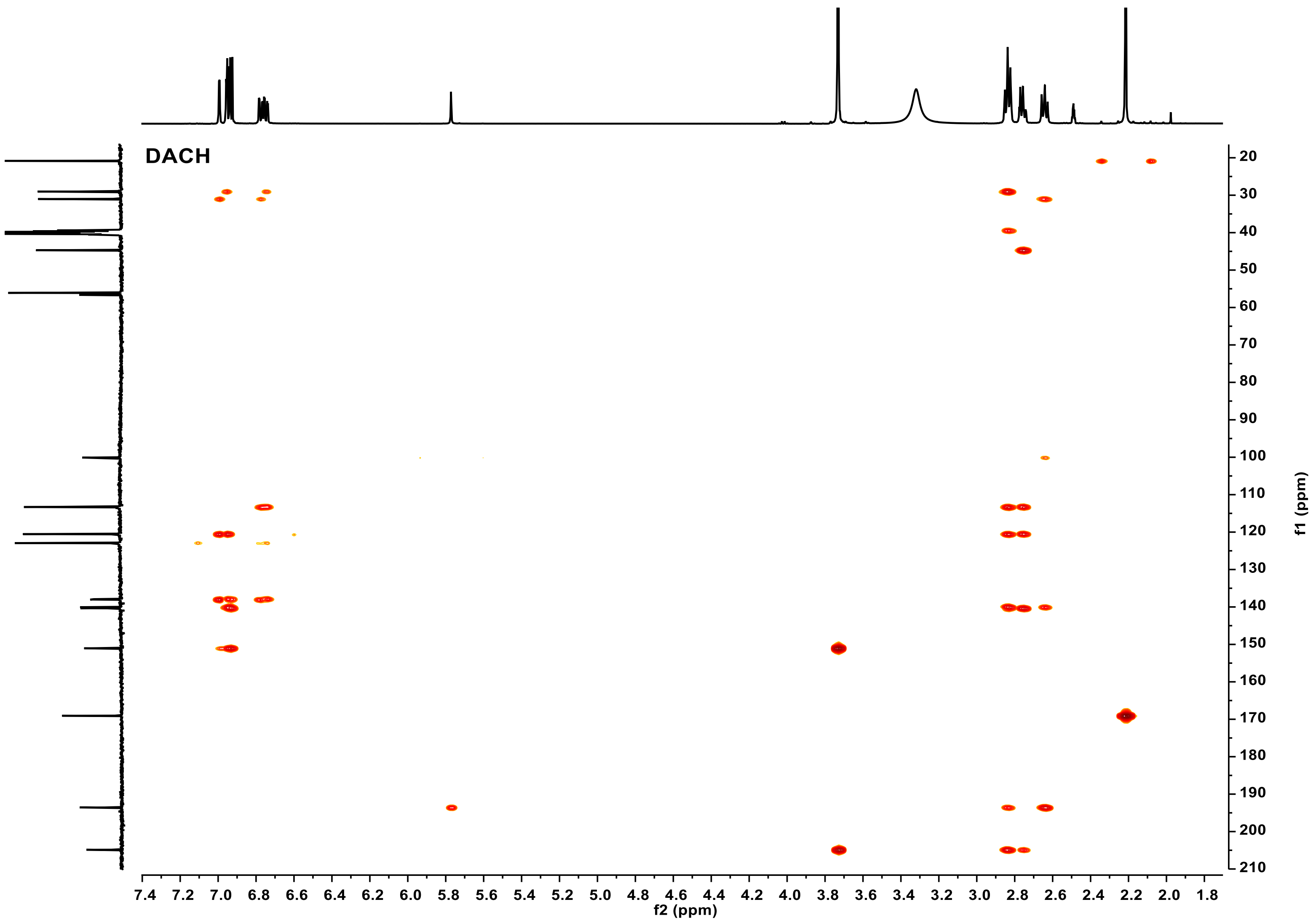

Figure S8. 500 MHz HMBC NMR spectrum of hydrogenated diacetyl-curcumin.

DiMeOC

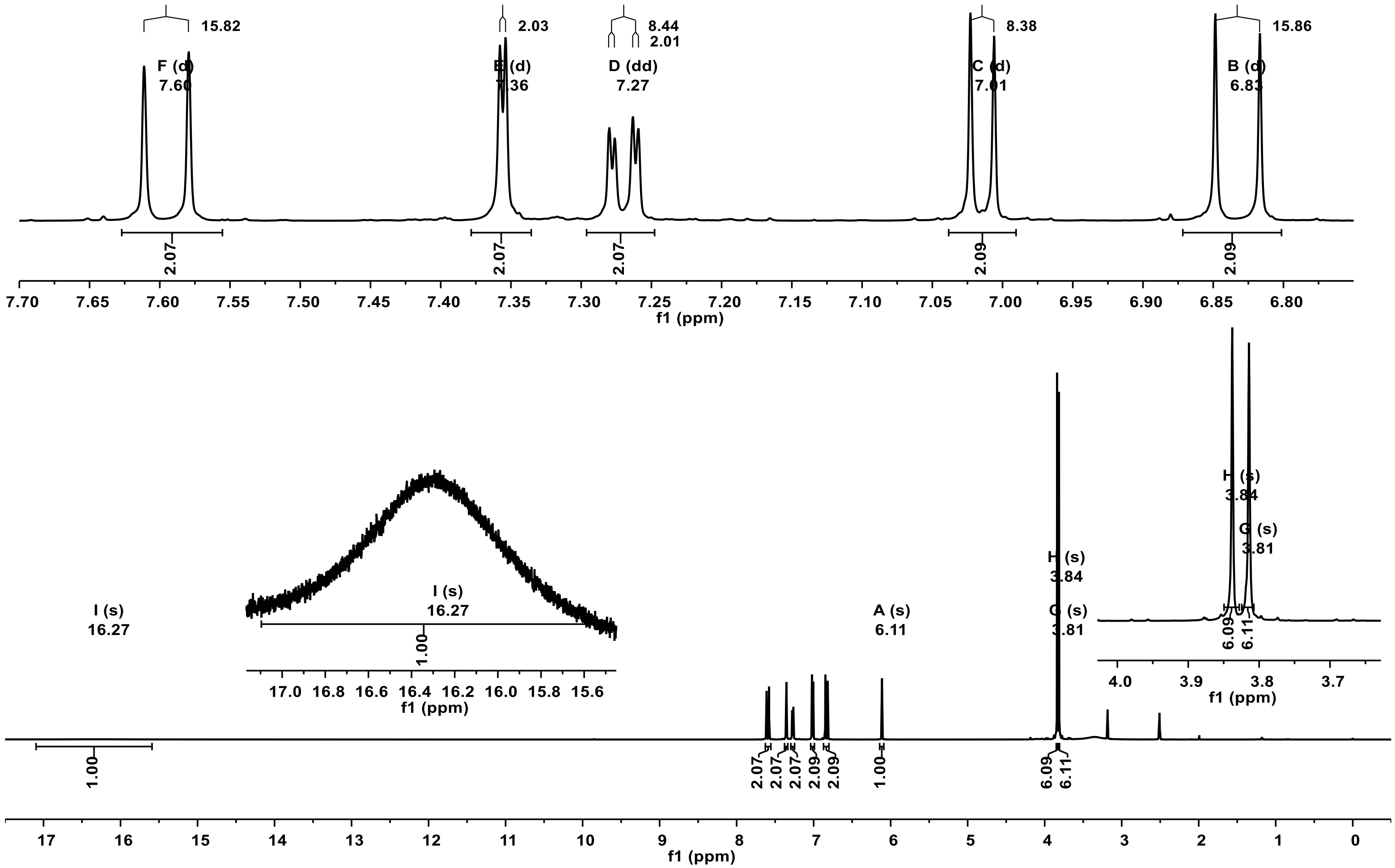

Figure S9. 500 MHz ^1H NMR spectrum of dimethoxy-curcumin.

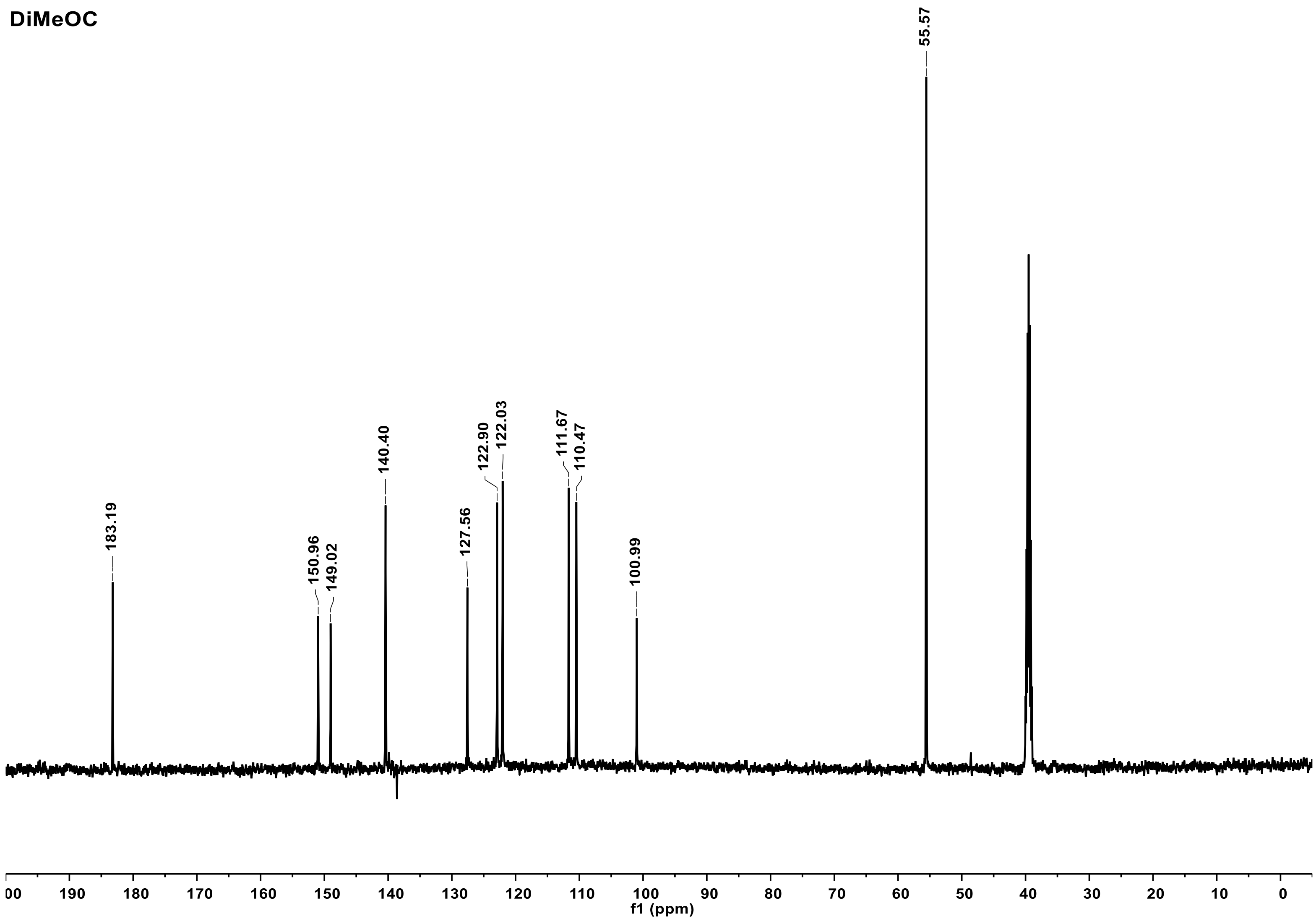

Figure S10. 125 MHz ^{13}C NMR spectrum of dimethoxy-curcumin.

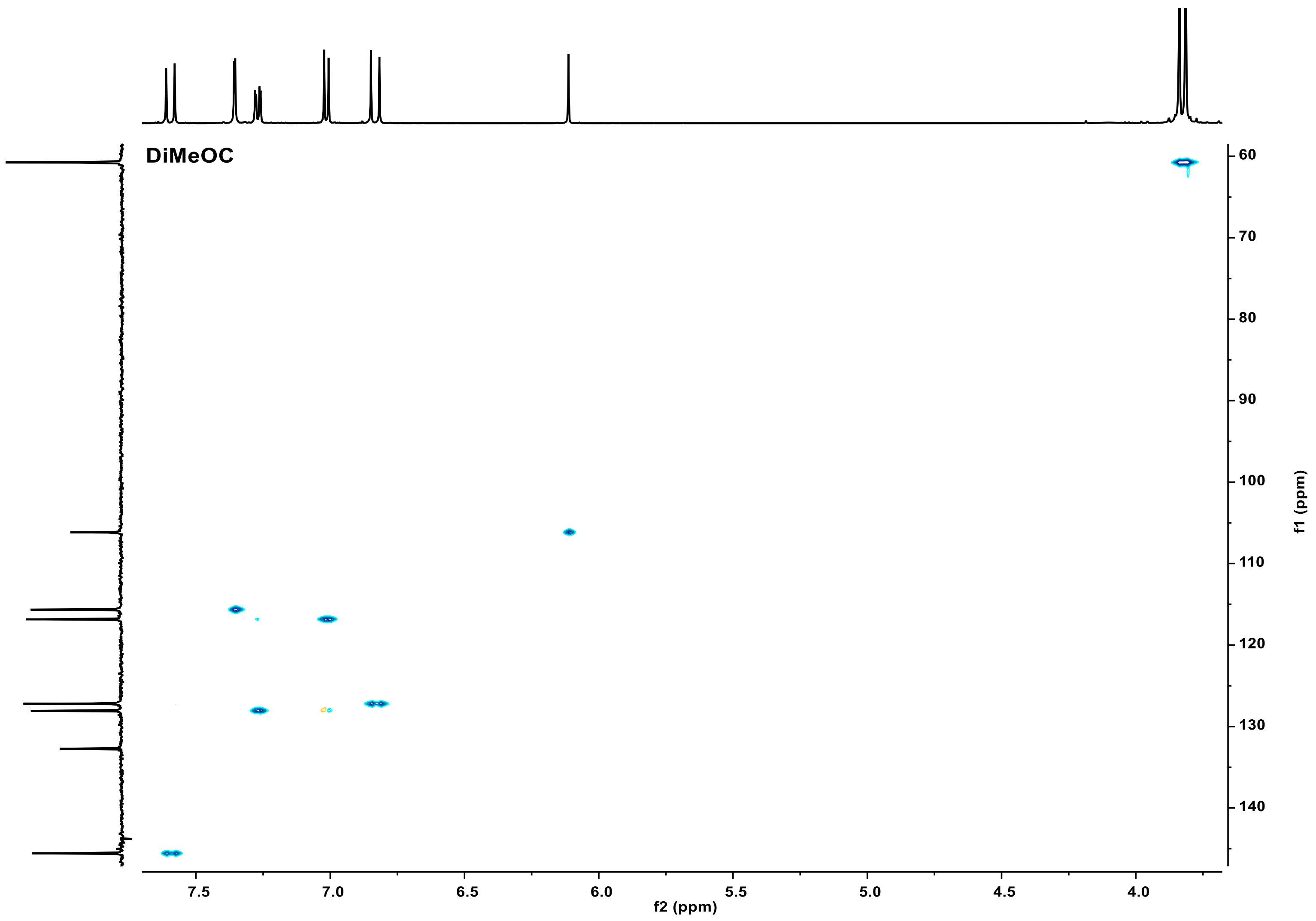

Figure S11. 500 MHz HSQC NMR spectrum of dimethoxy-curcumin.

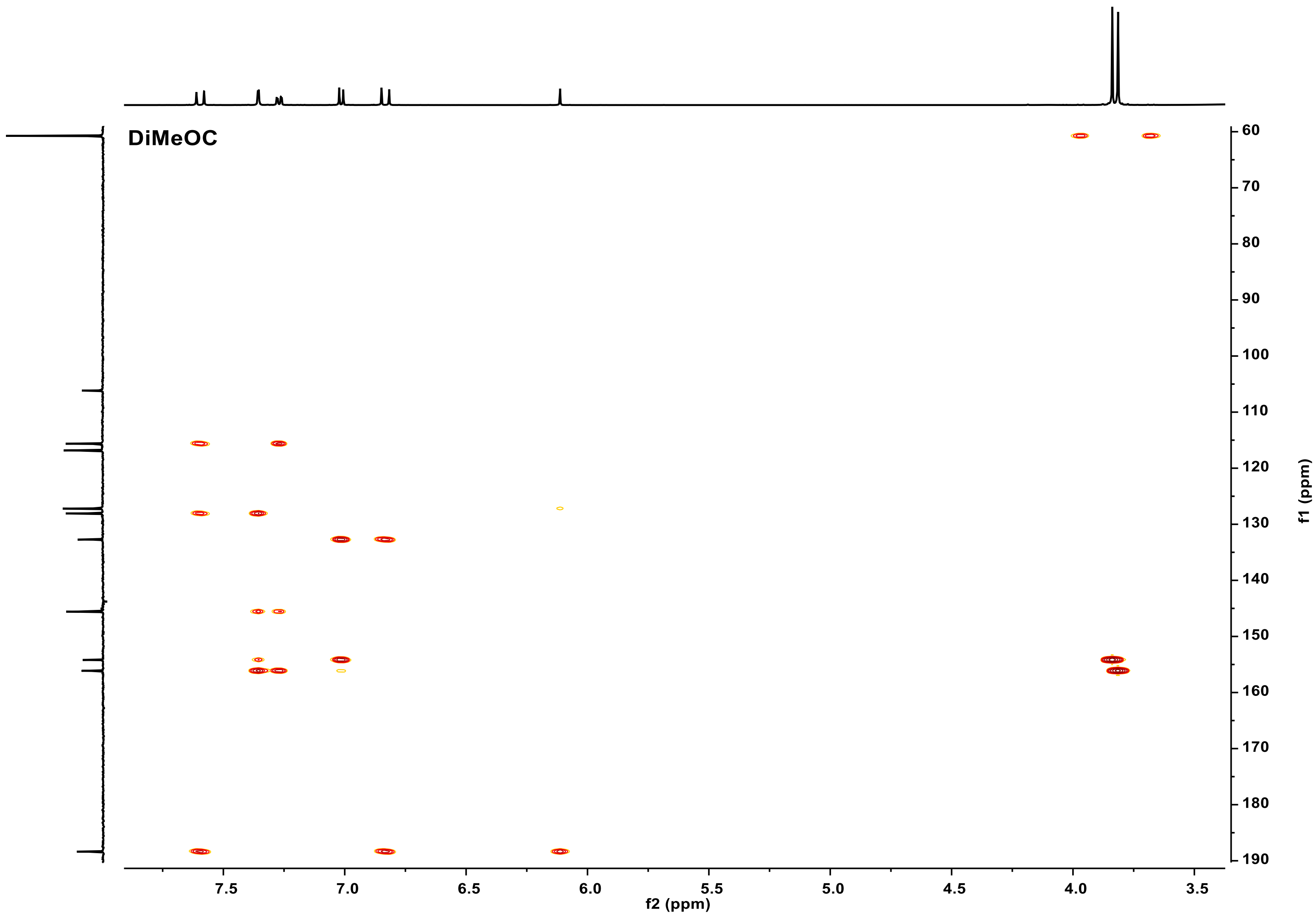

Figure S12. 500 MHz HMBC NMR spectrum of dimethoxy-curcumin.

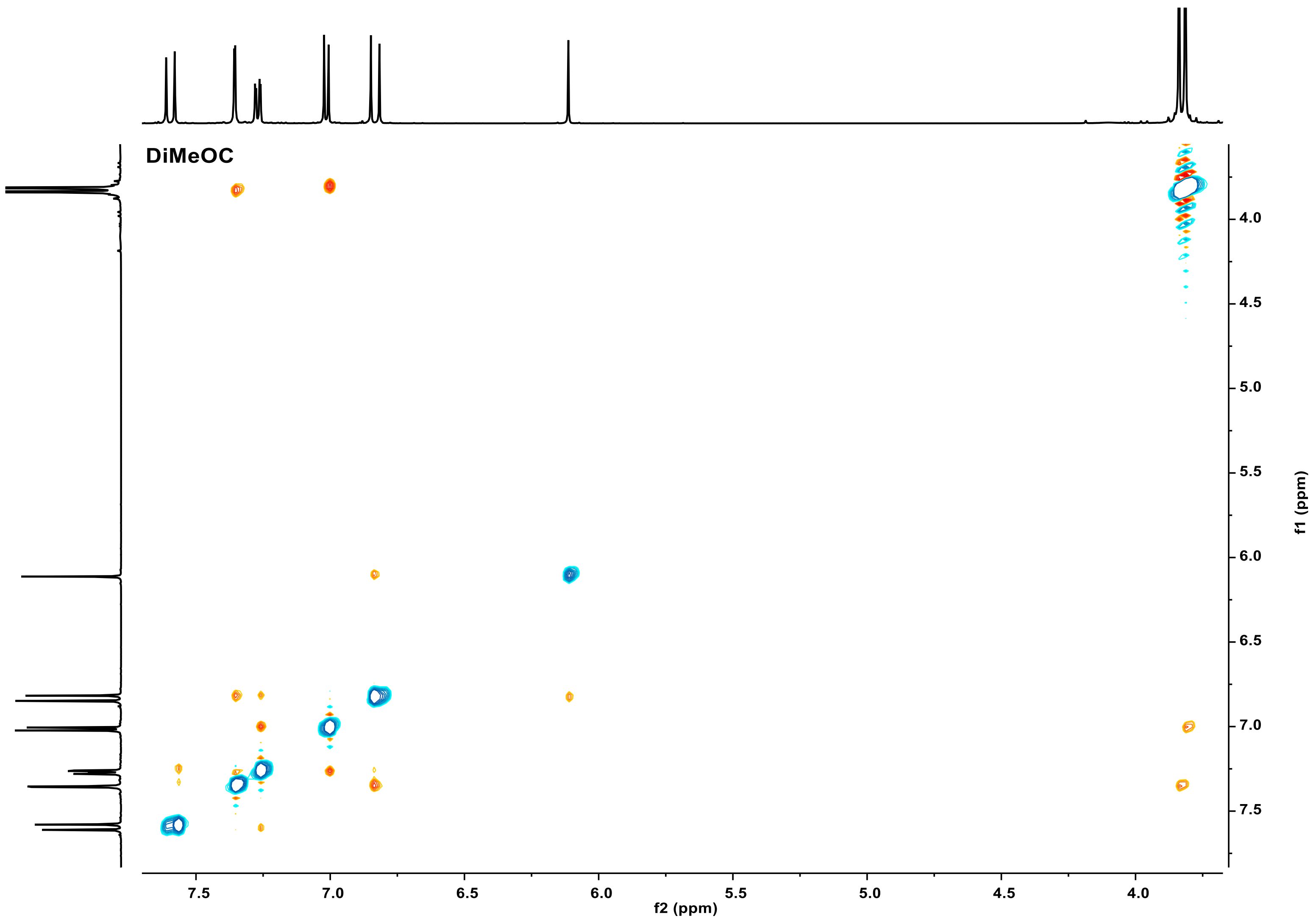

Figure S13. 500 MHz ROESY NMR spectrum of dimethoxy-curcumin.

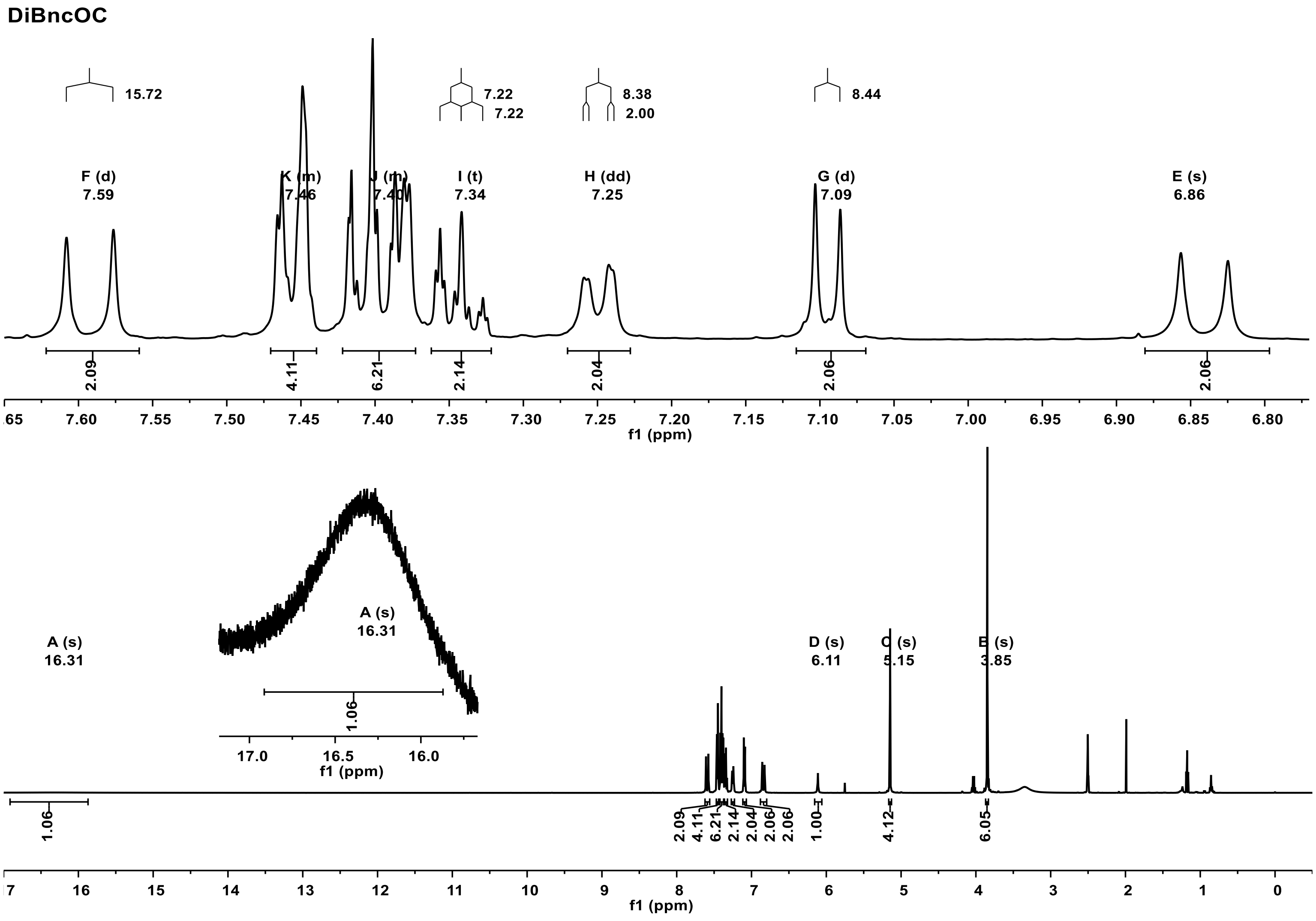

Figure S14. 500 MHz ^{1}H NMR spectrum of dibenzyl-curcumin.

DiBncOC

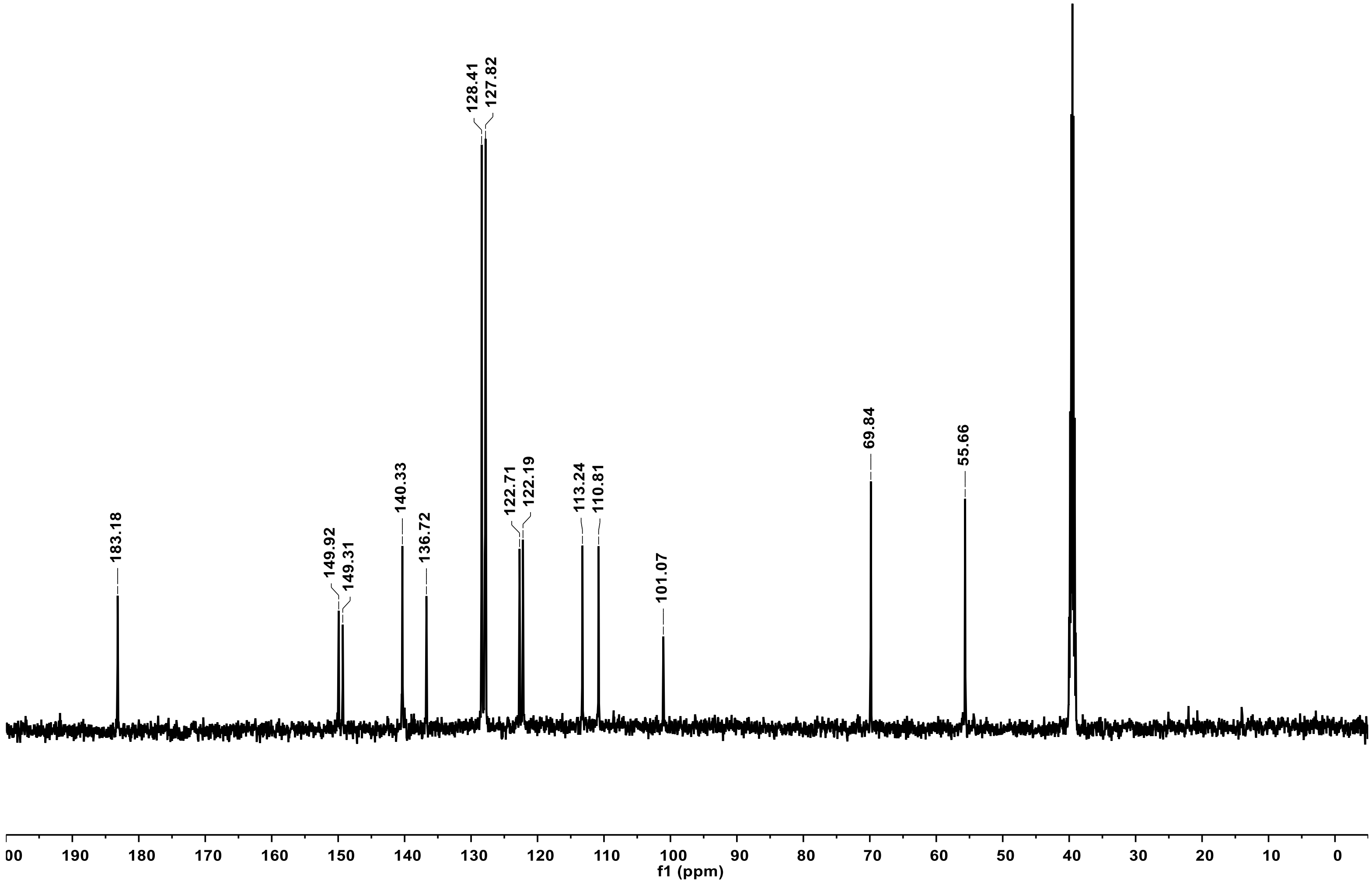

Figure S15. 125 MHz ^{13}C NMR spectrum of dibenzyl-curcumin.

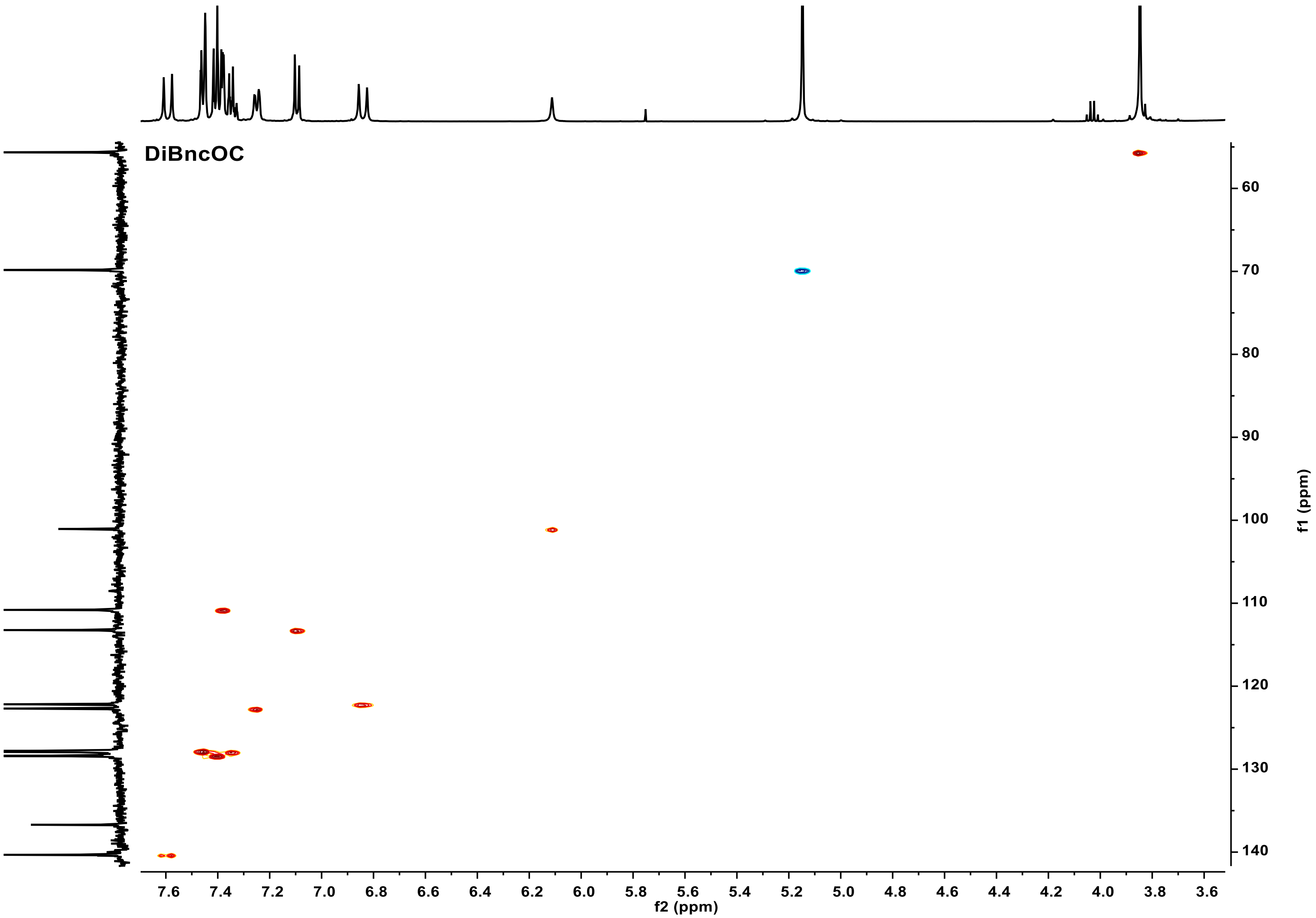

Figure S16. 500 MHz HSQC NMR of dibenzyl-curcumin.

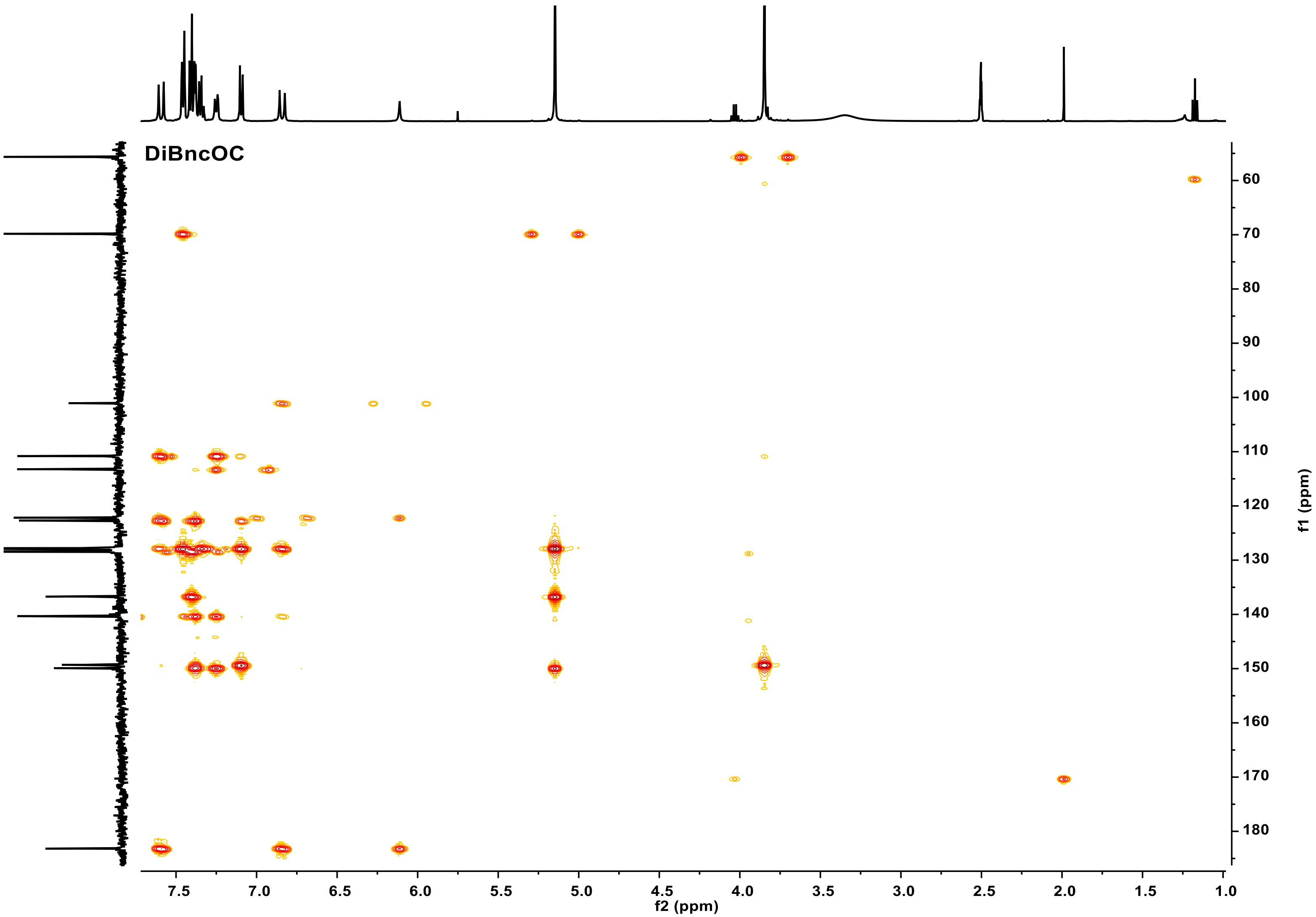

Figura S17. 500 MHz HMBC NMR of dibenzyl-curcumin.

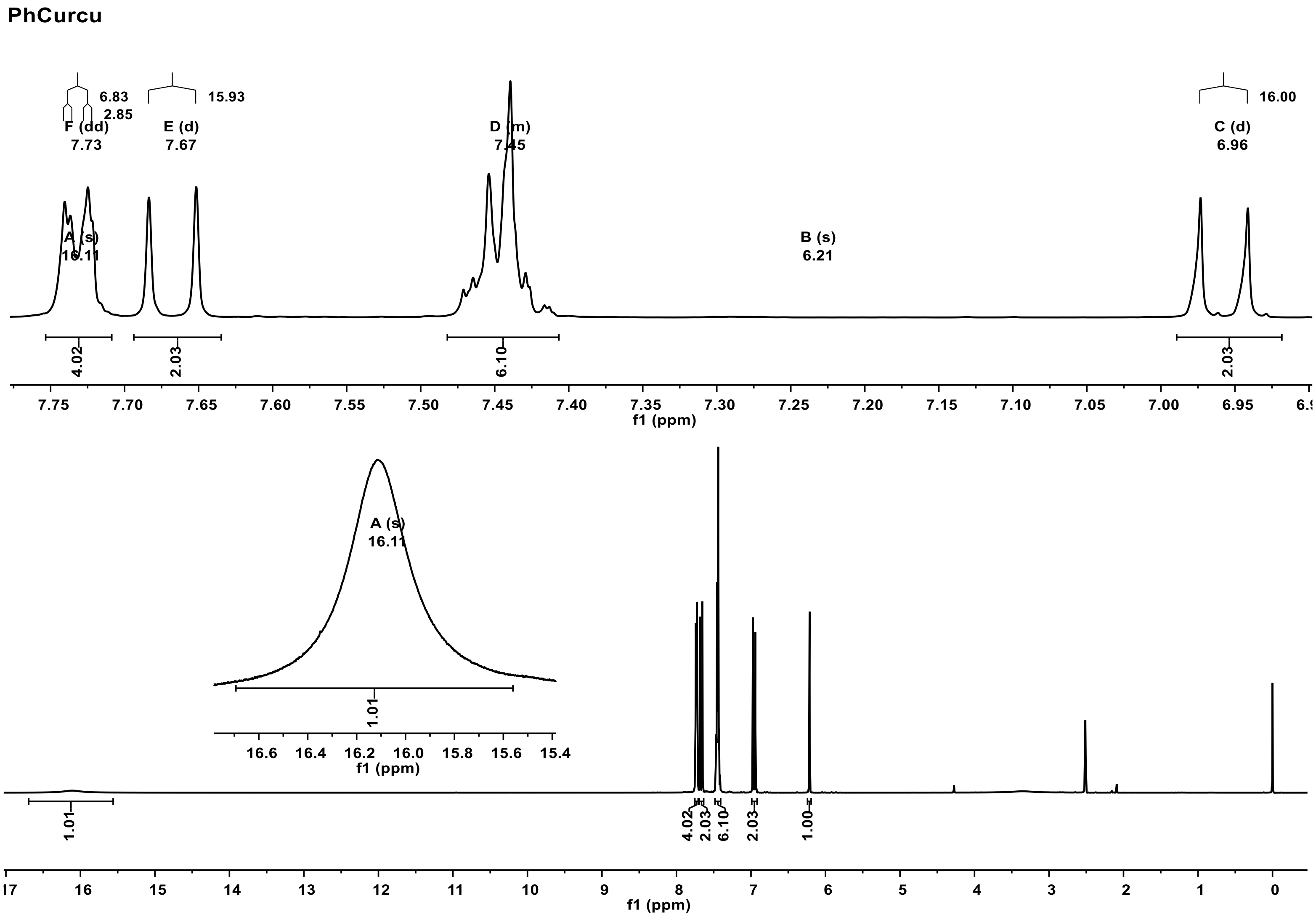

Figure S18. 500 MHz ^{1}H NMR spectrum of diphenyl-curcumin.

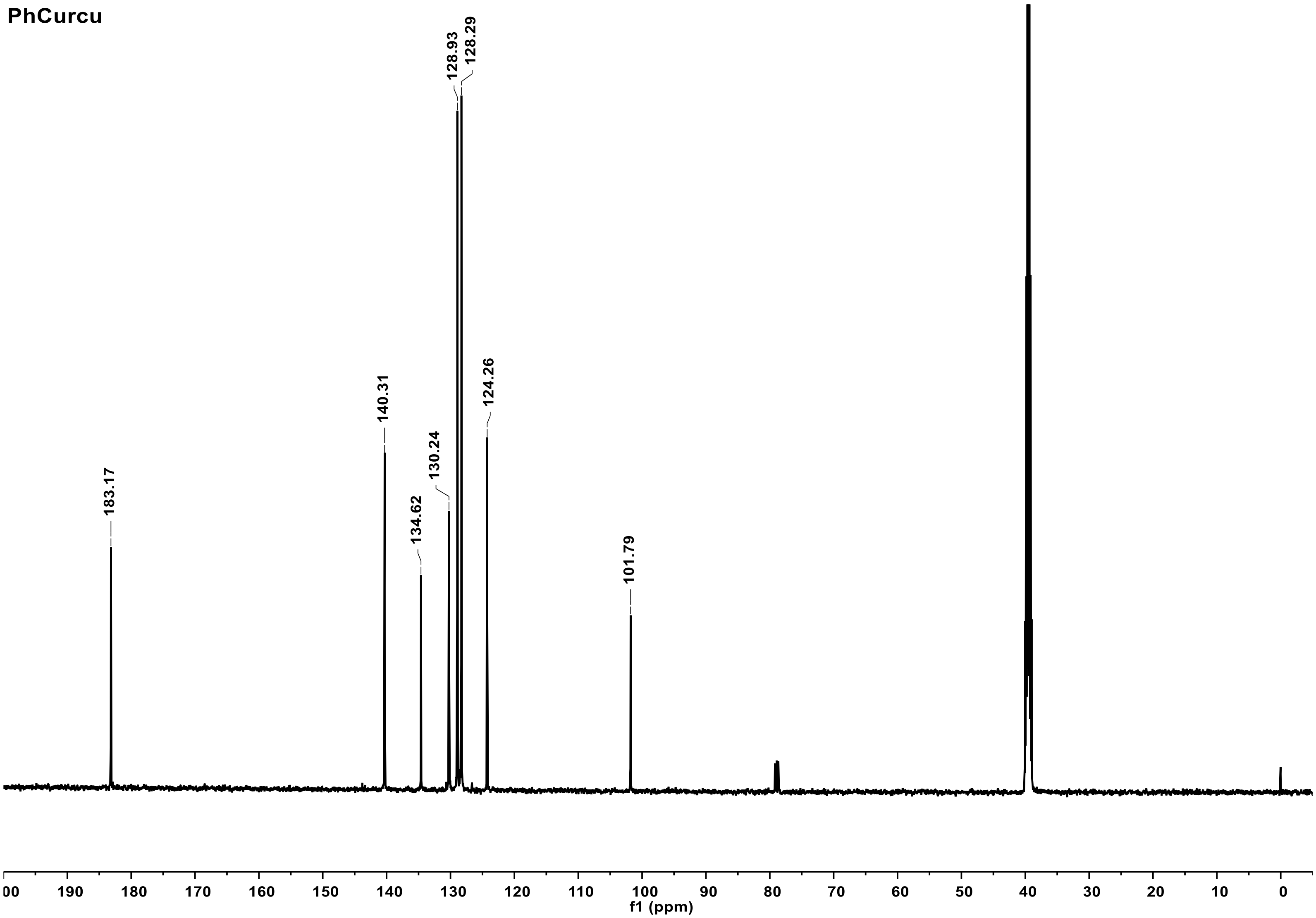

Figure S19. 125 MHz ^{13}C NMR spectrum of diphenyl-curcumin.

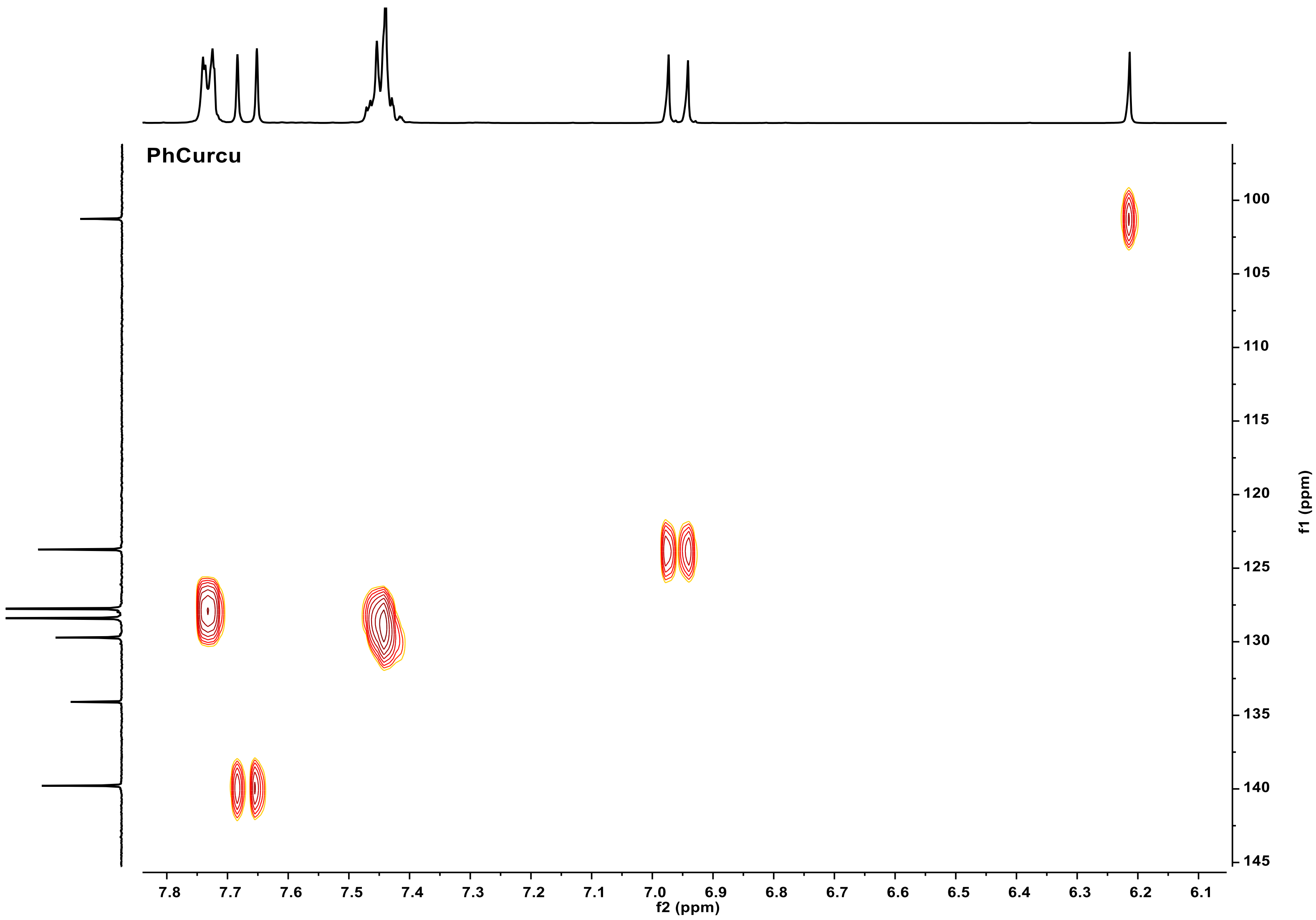

Figure S20. 500 MHz HSQC NMR of diphenyl-curcumin.

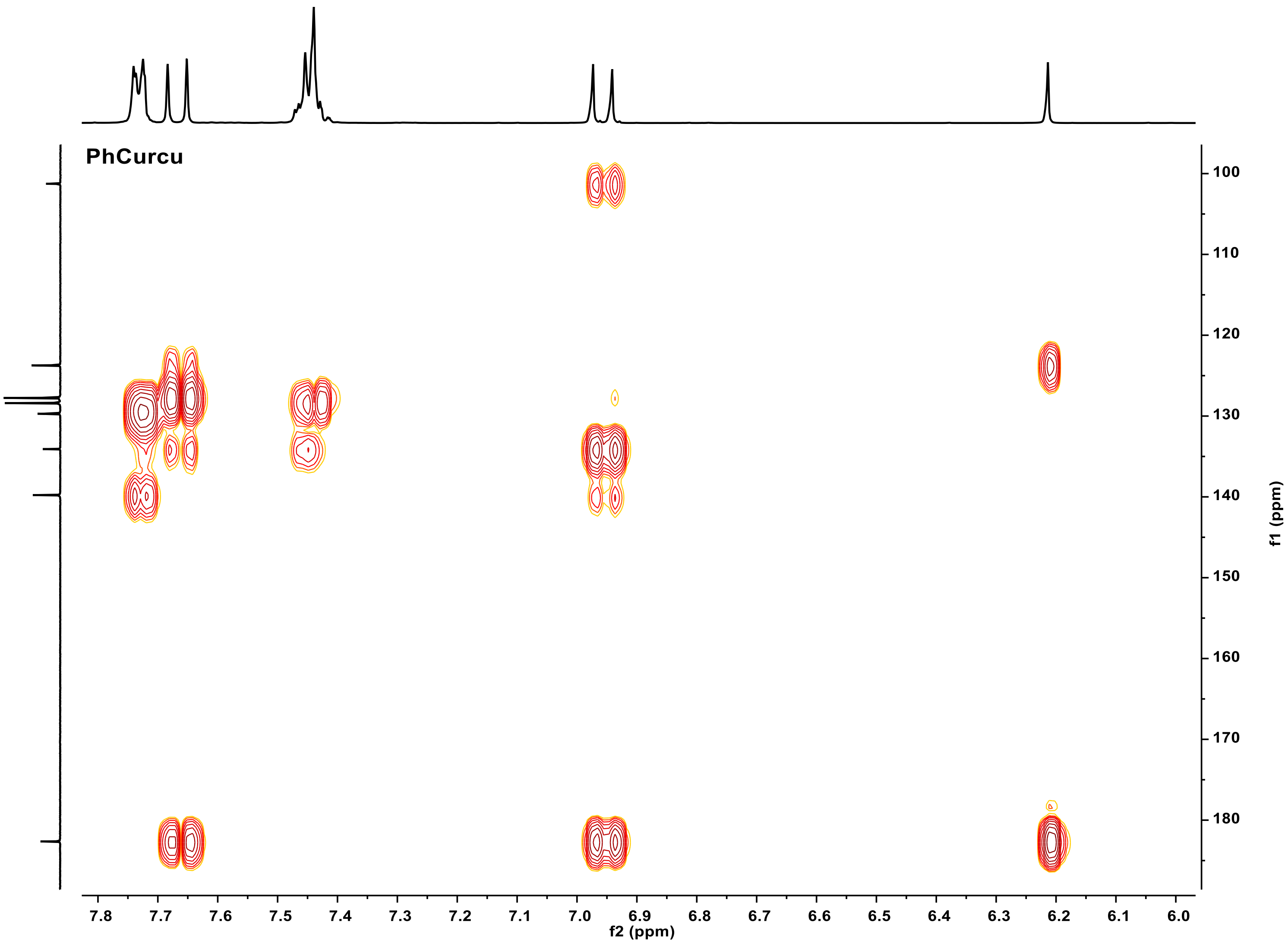

Figure S21. 500 MHz HMBC NMR of diphenyl-curcumin.

DAC-Cu

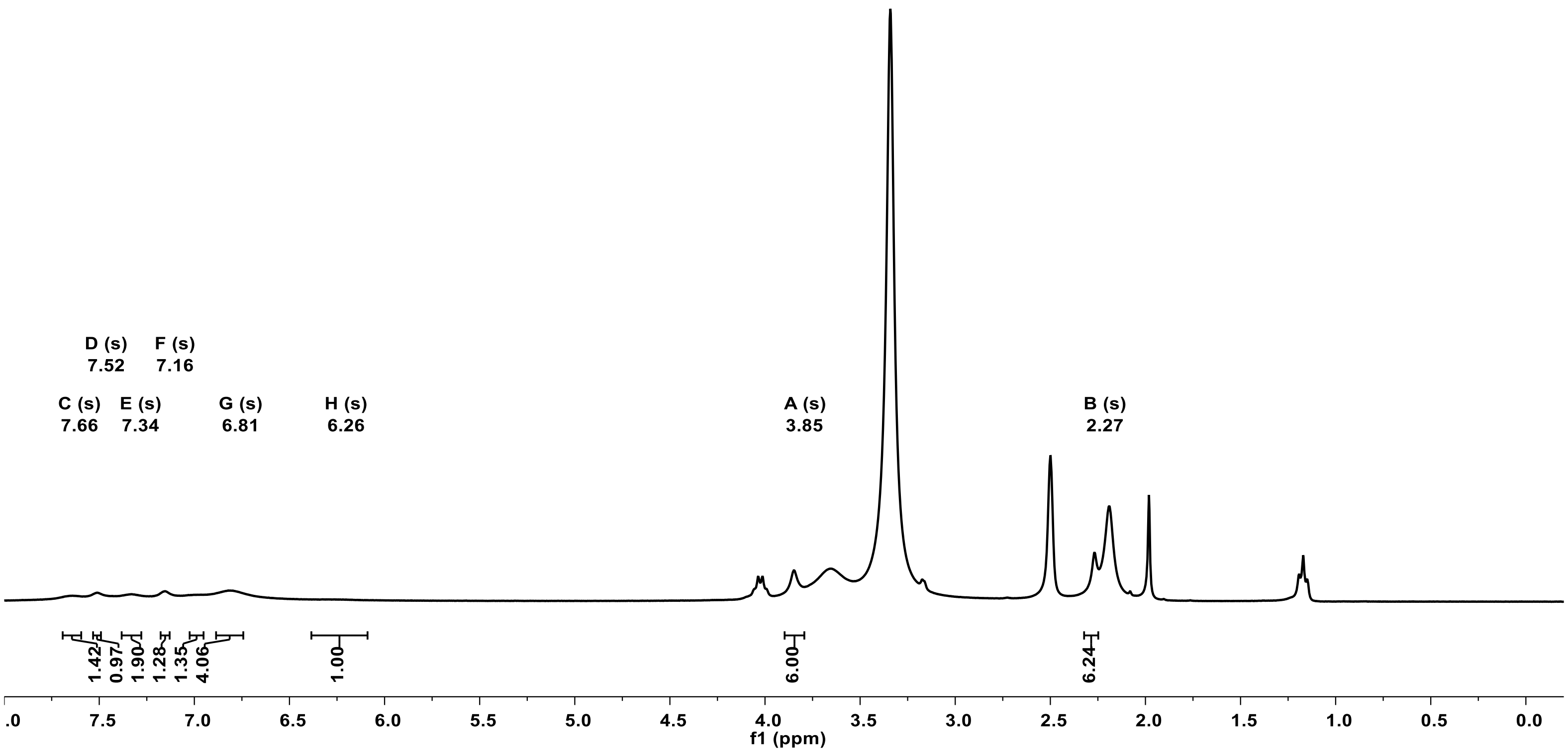

Figure S22. 500 MHz ^{1}H NMR spectrum of diacetyl-curcumin with Cu (II).

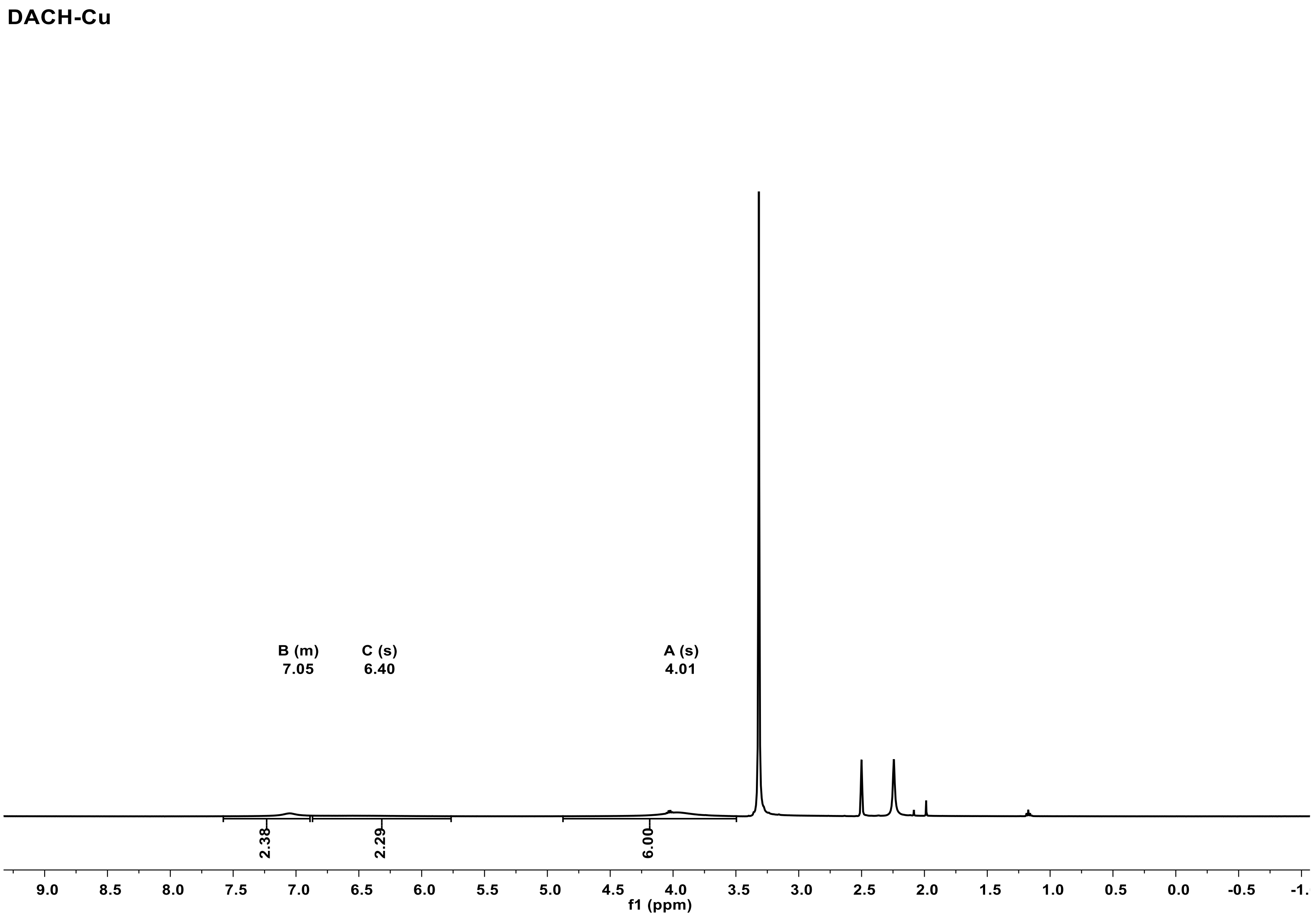

Figure S23. 500 MHz ^{1}H NMR spectrum of hydrogenated diacetyl-curcumin with Cu (II).

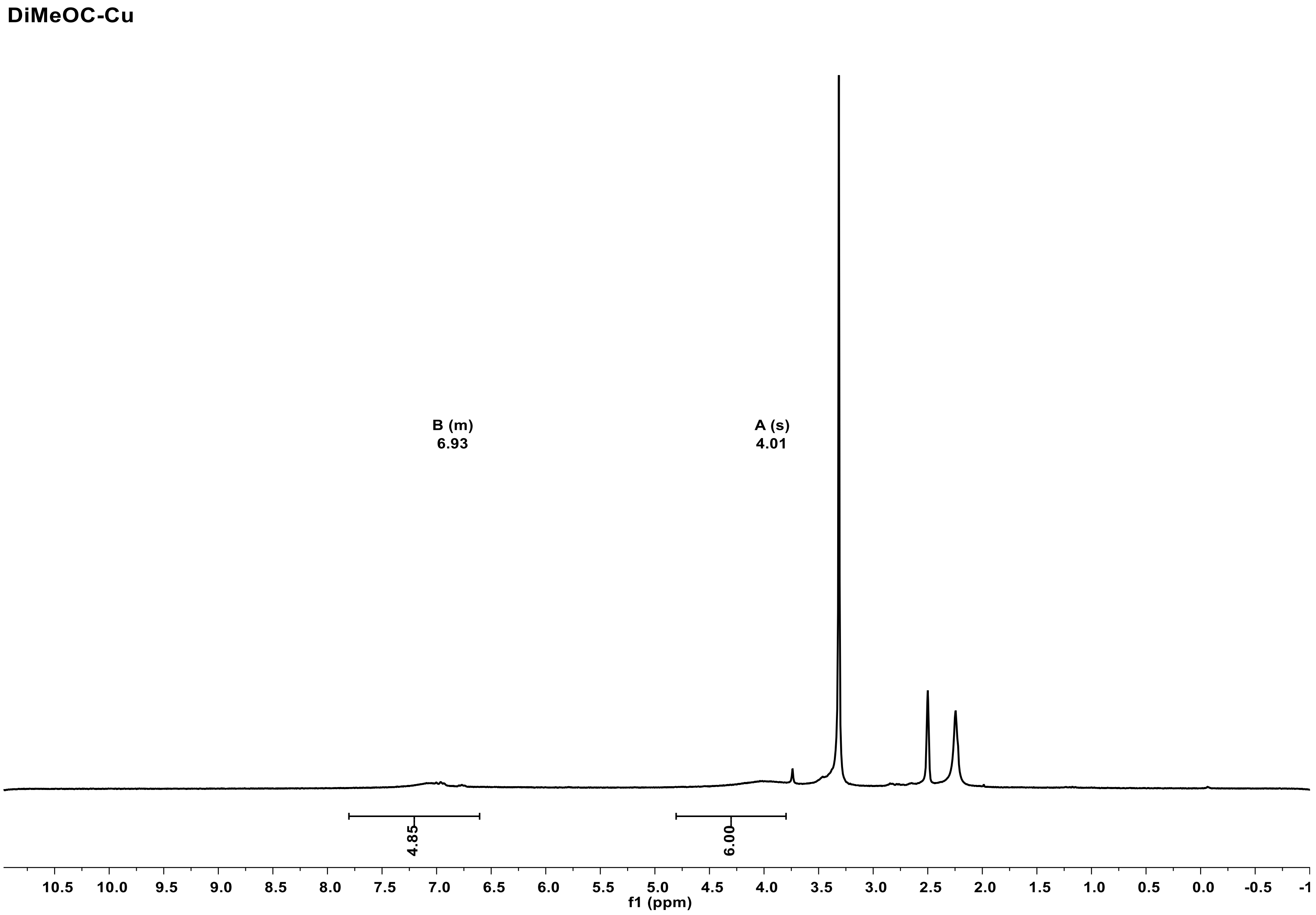

Figure S24. 500 MHz ^{1}H NMR spectrum of dimethoxy-curcumin with Cu (II).

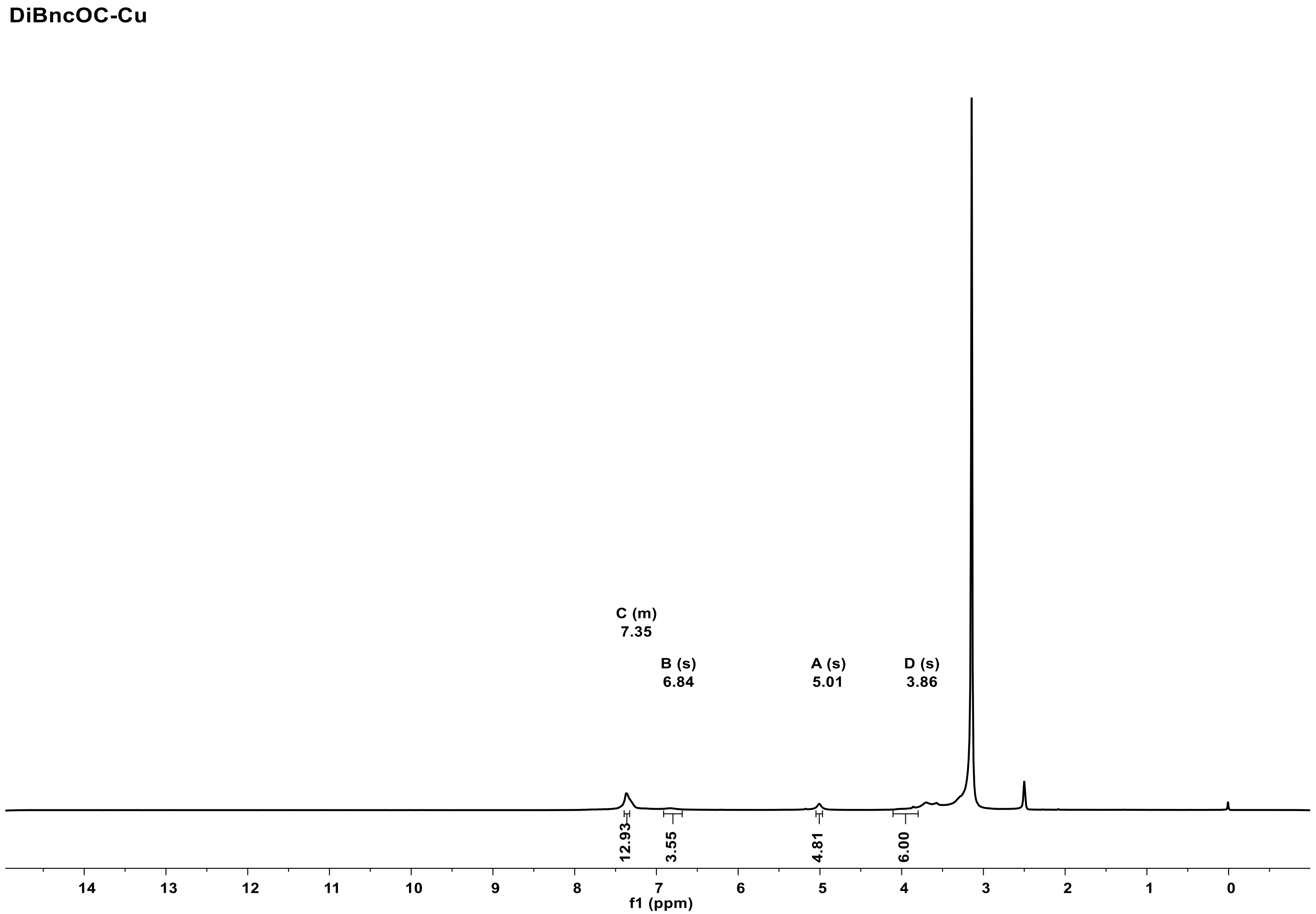

Figure S25. 500 MHz ^{1}H NMR spectrum of dibenzyl-curcumin with Cu (II).

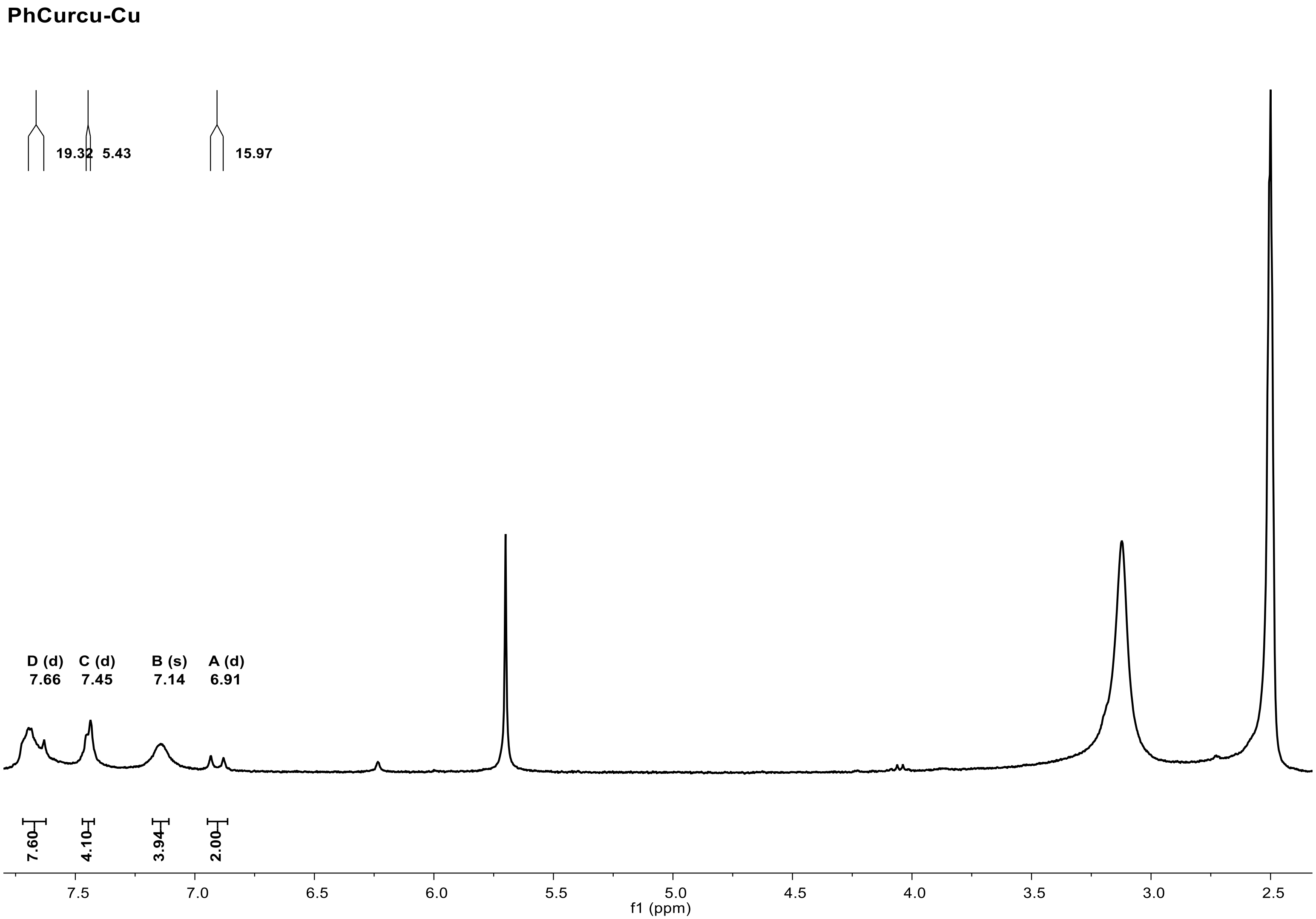

Figure S26. 500 MHz ^{1}H NMR spectrum of diphenyl-curcumin with Cu (II).

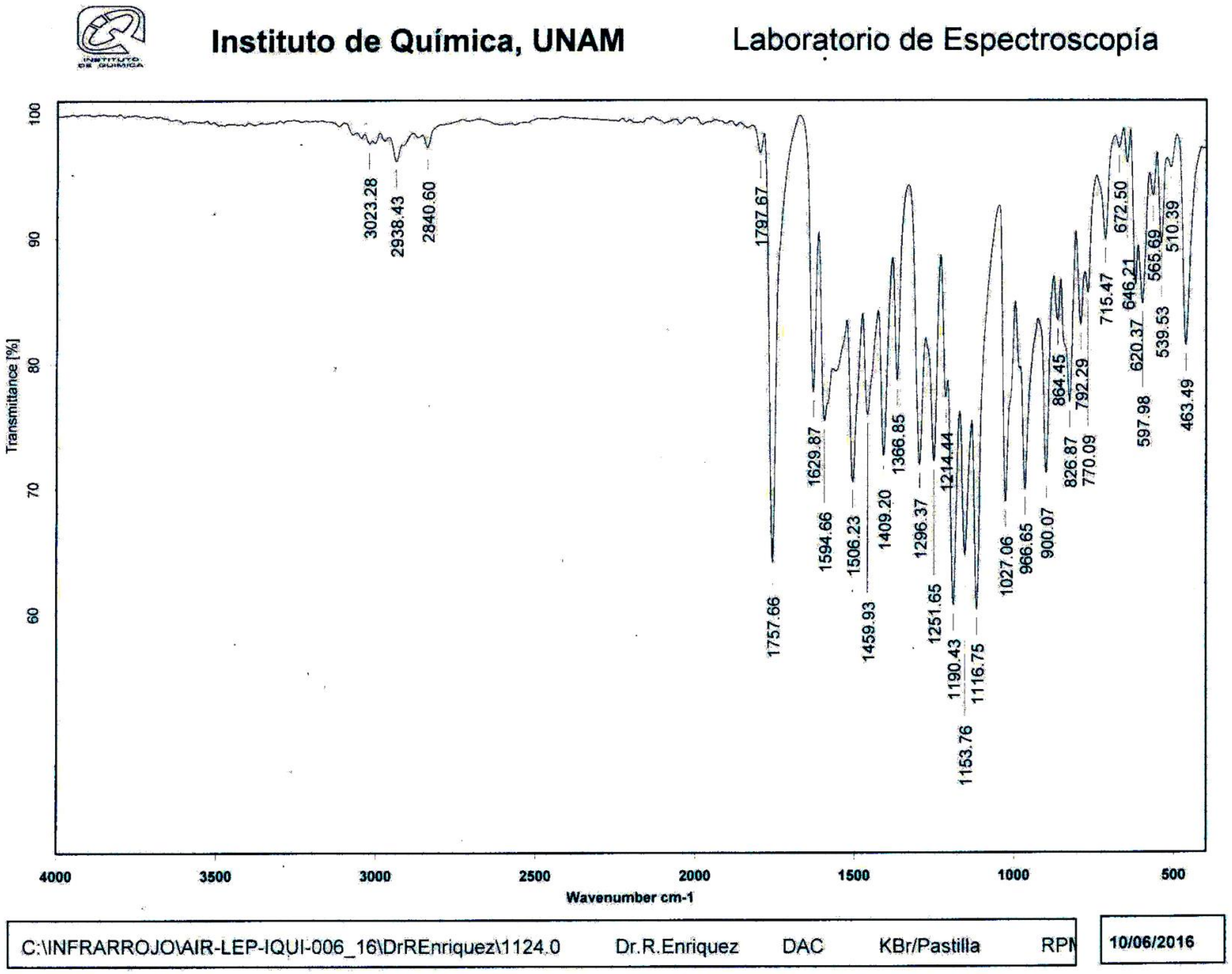

Figure S27. IR Spectrum of diacetyl-curcumin.

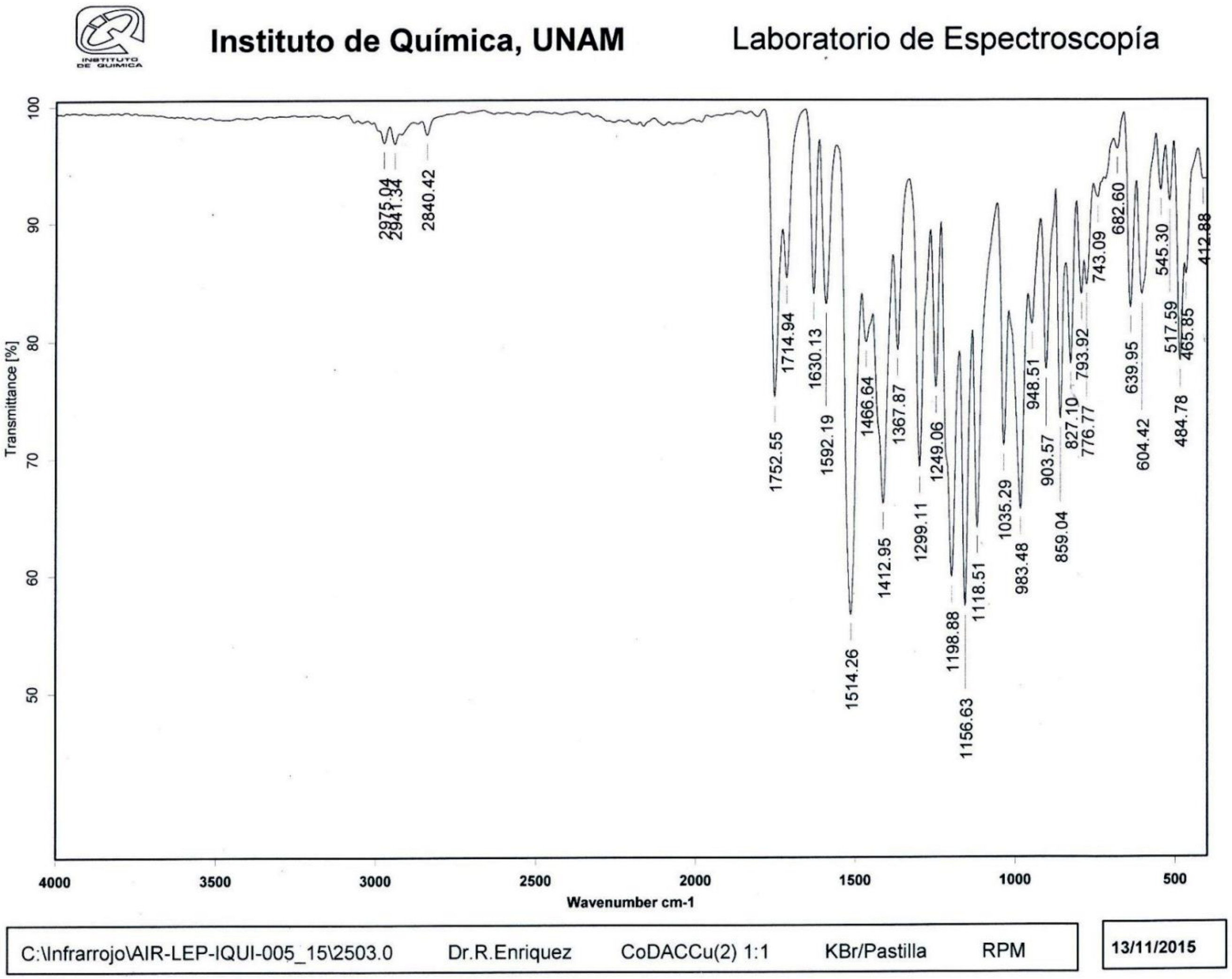

Figure S28. IR Spectrum of diacetyl-curcumin with Cu (II).

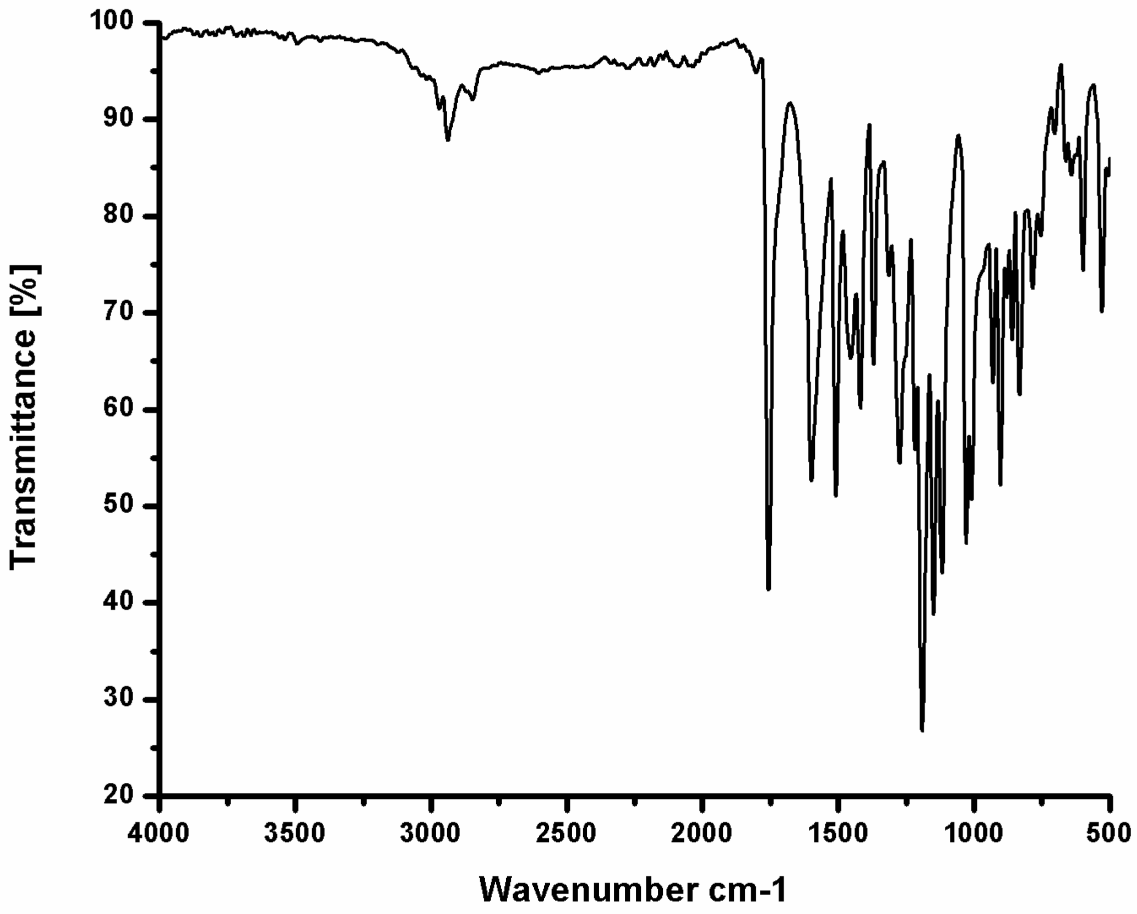

Figure S29. IR Spectrum of hydrogenated diacetyl -curcumin.

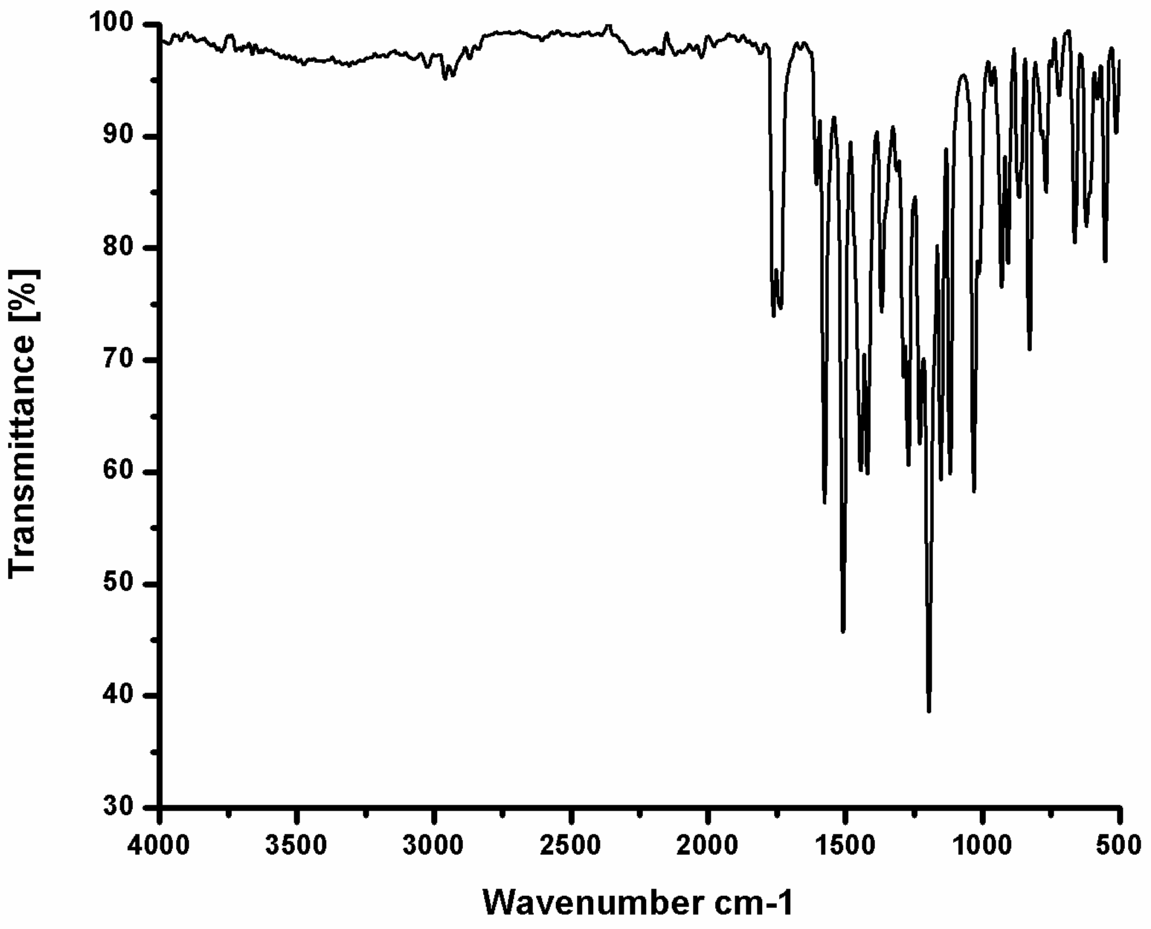

Figure S30. IR Spectrum of hydrogenated diacetyl-curcumin with Cu (II).

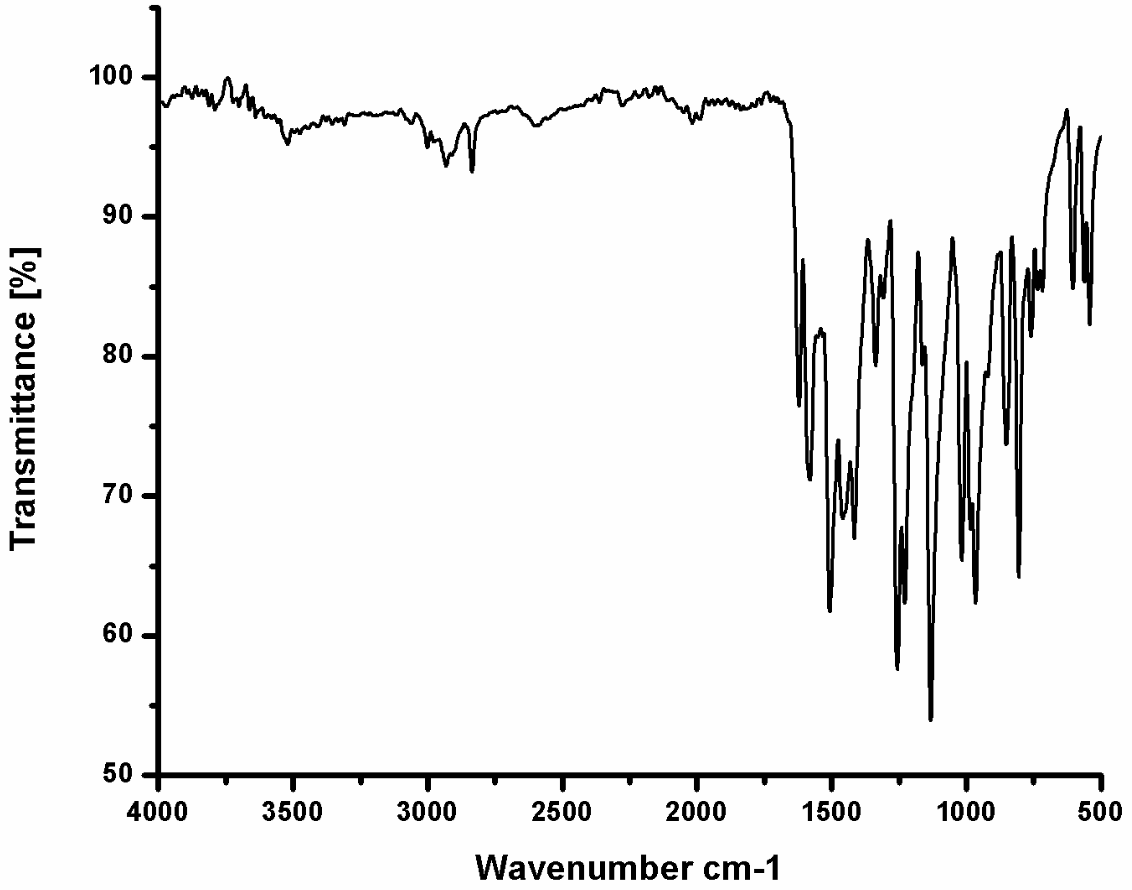

Figure S31. IR Spectrum of dimethoxy-curcumin.

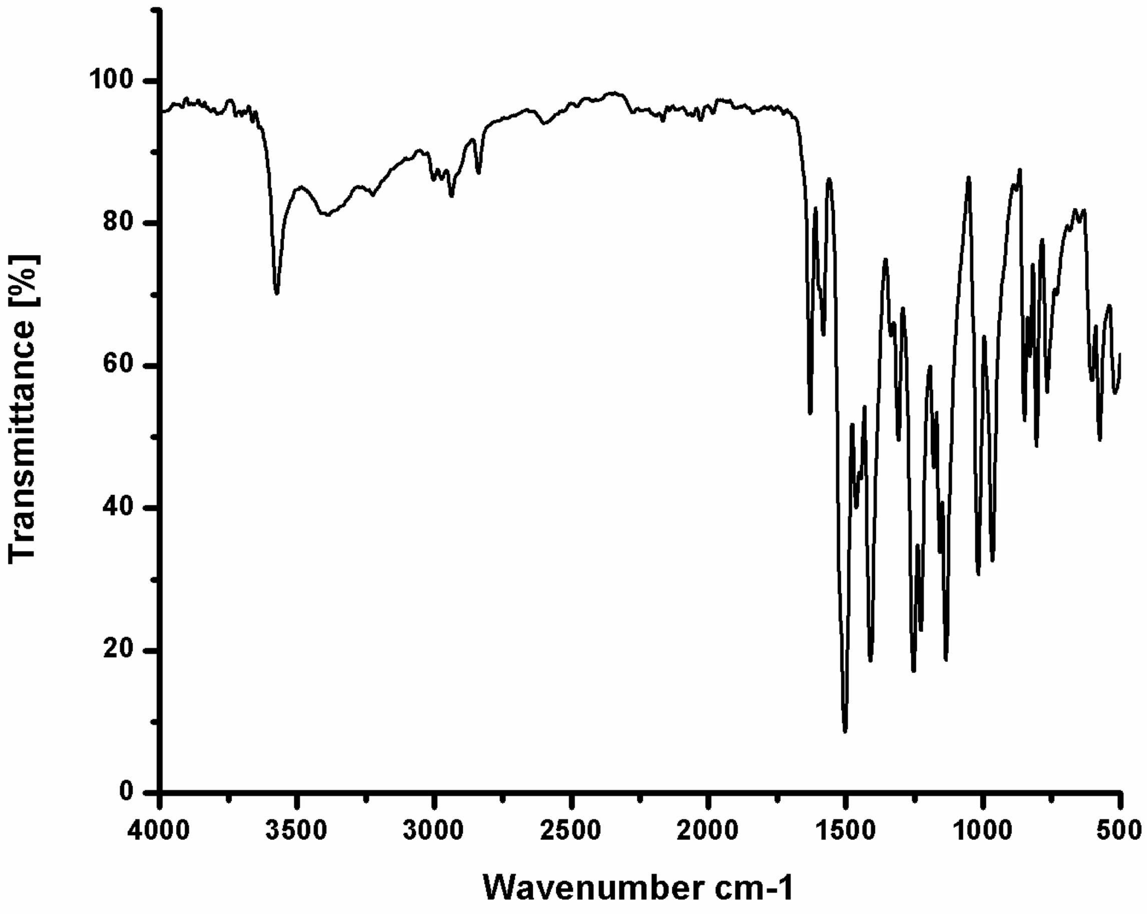

Figure S32. IR Spectrum of dimethoxy-curcumin with Cu (II).

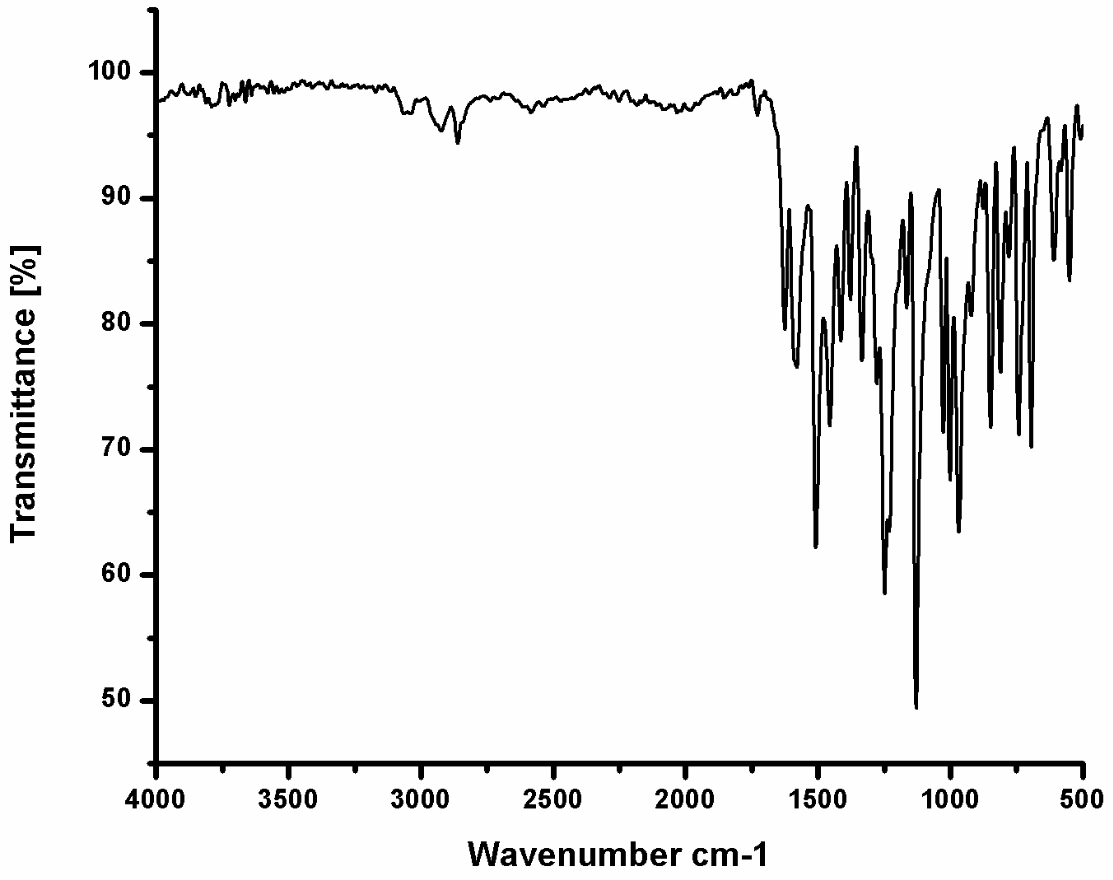

Figure S33. IR Spectrum of dibenzyl-curcumin.

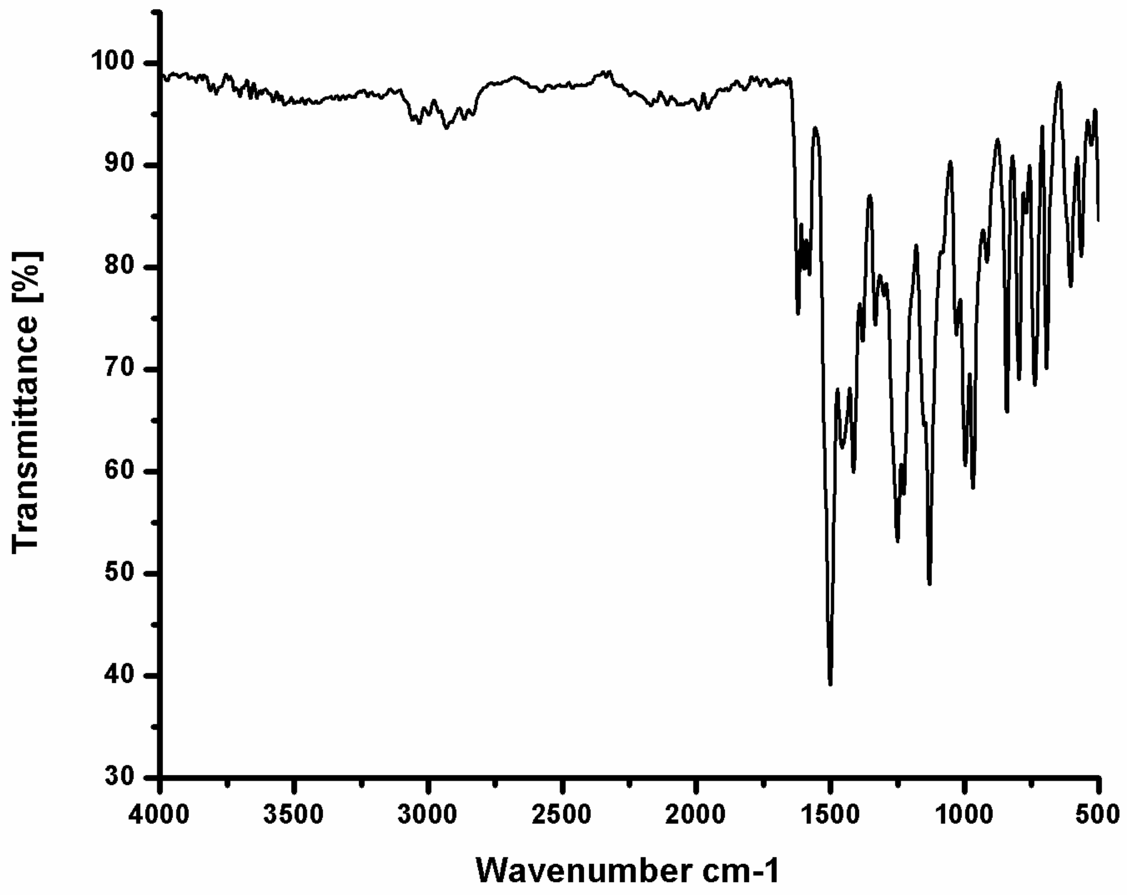

Figure S34. IR Spectrum of dibenzyl-curcumin with Cu (II).

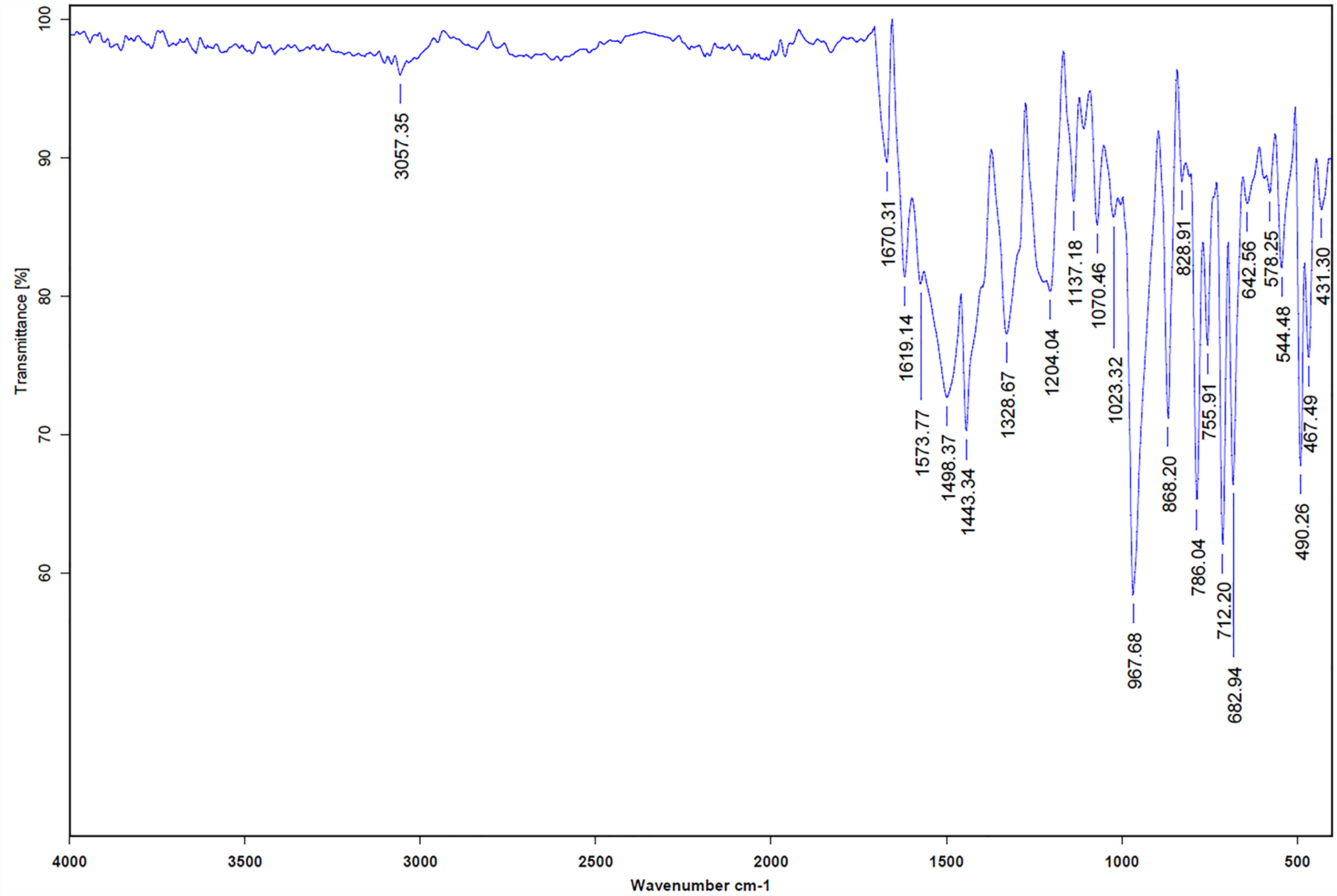

Figure S35. IR Spectrum of diphenyl-curcumin.

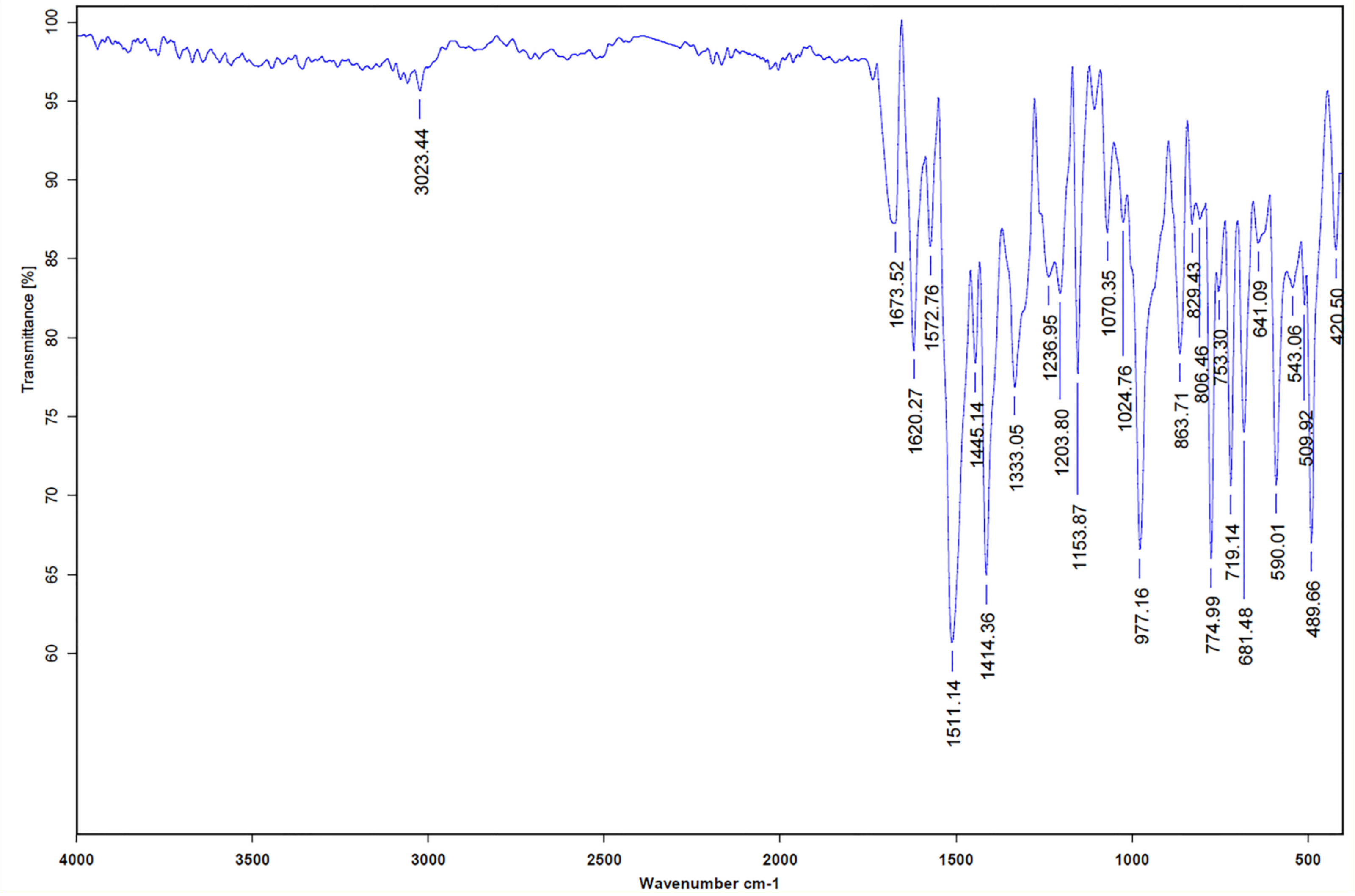

Figure S36. IR Spectrum of diphenyl-curcumin with Cu (II).

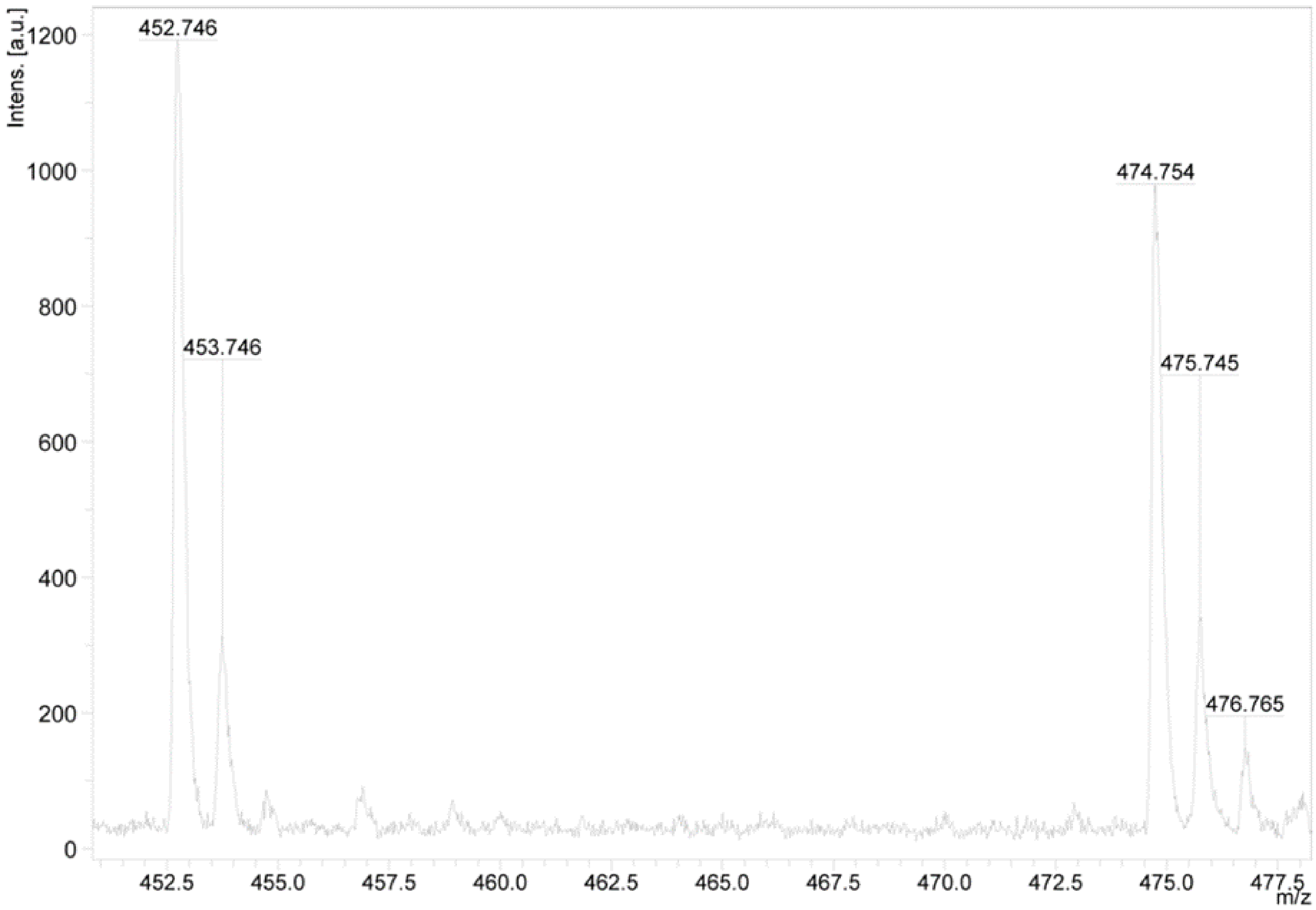

Figura S42. Mass Spectrum of diacetyl-curcumin.

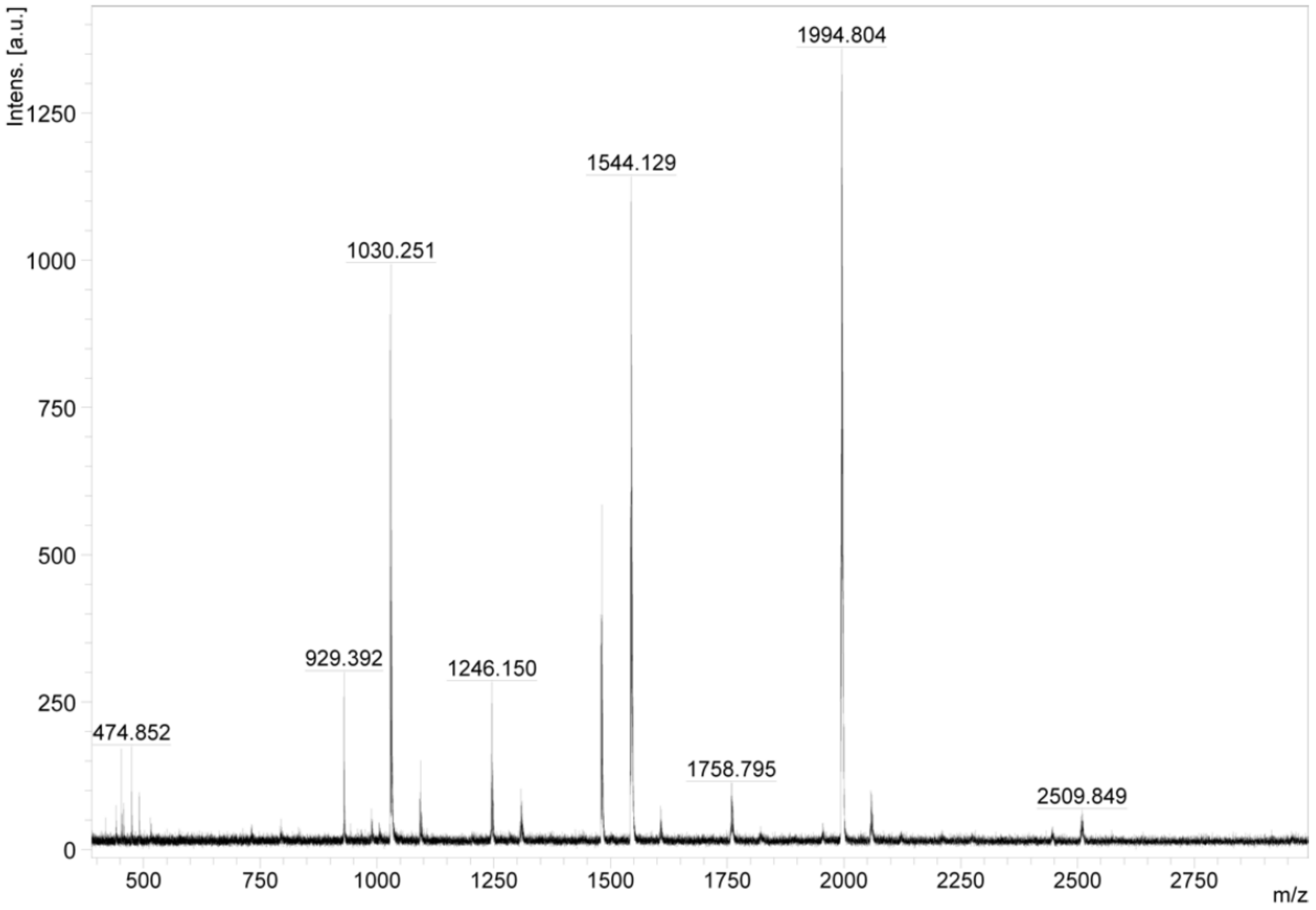

Figura S45. Mass Spectrum of diacetyl-curcumin.with Cu (II).

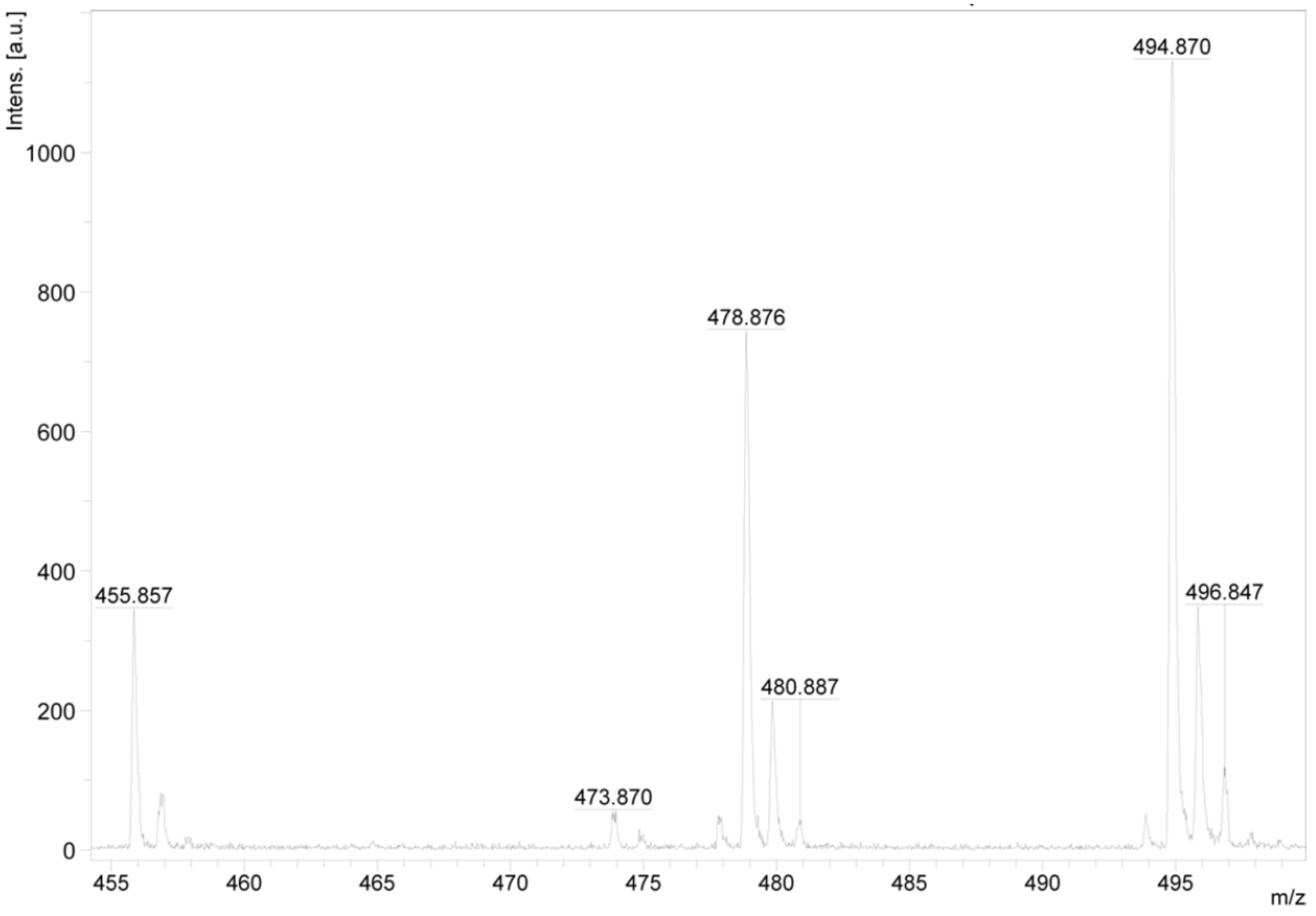

Figura S42. Mass Spectrum of hydrogenated diacetyl -curcumin.

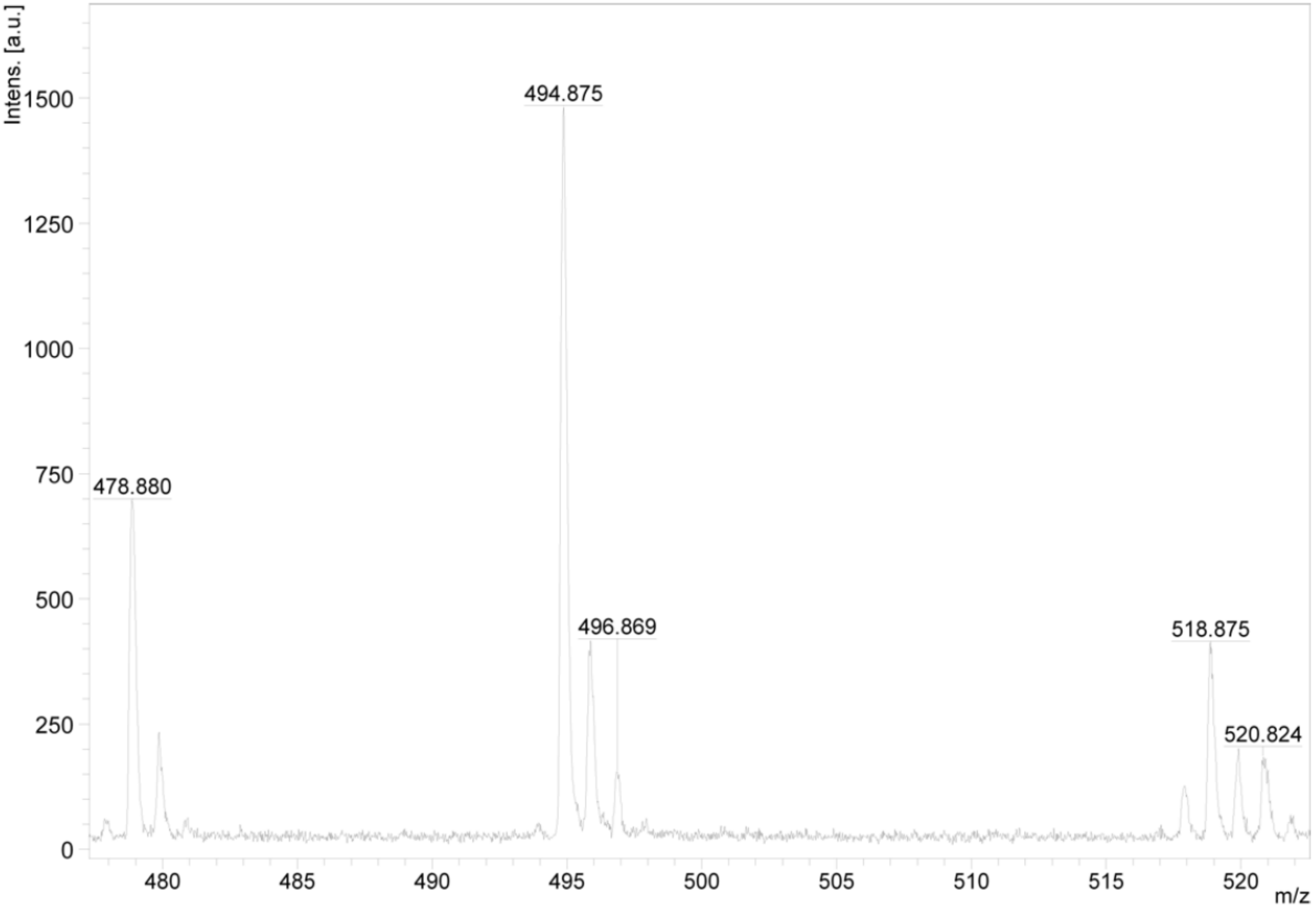

Figura S45. Mass Spectrum of hydrogenated diacetyl -curcumin.with Cu (II).

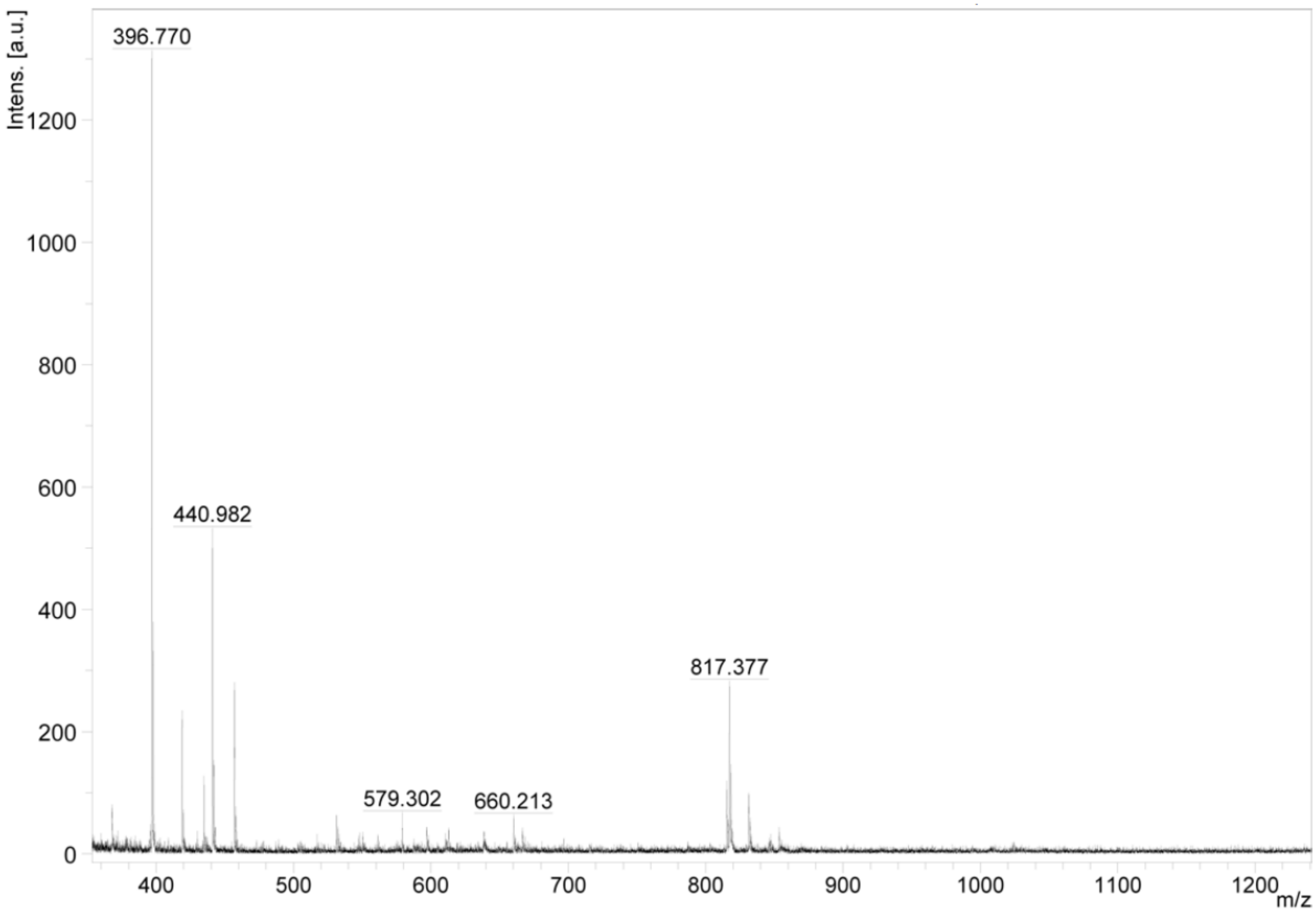

Figura S42. Mass Spectrum of dimethoxy-curcumin.

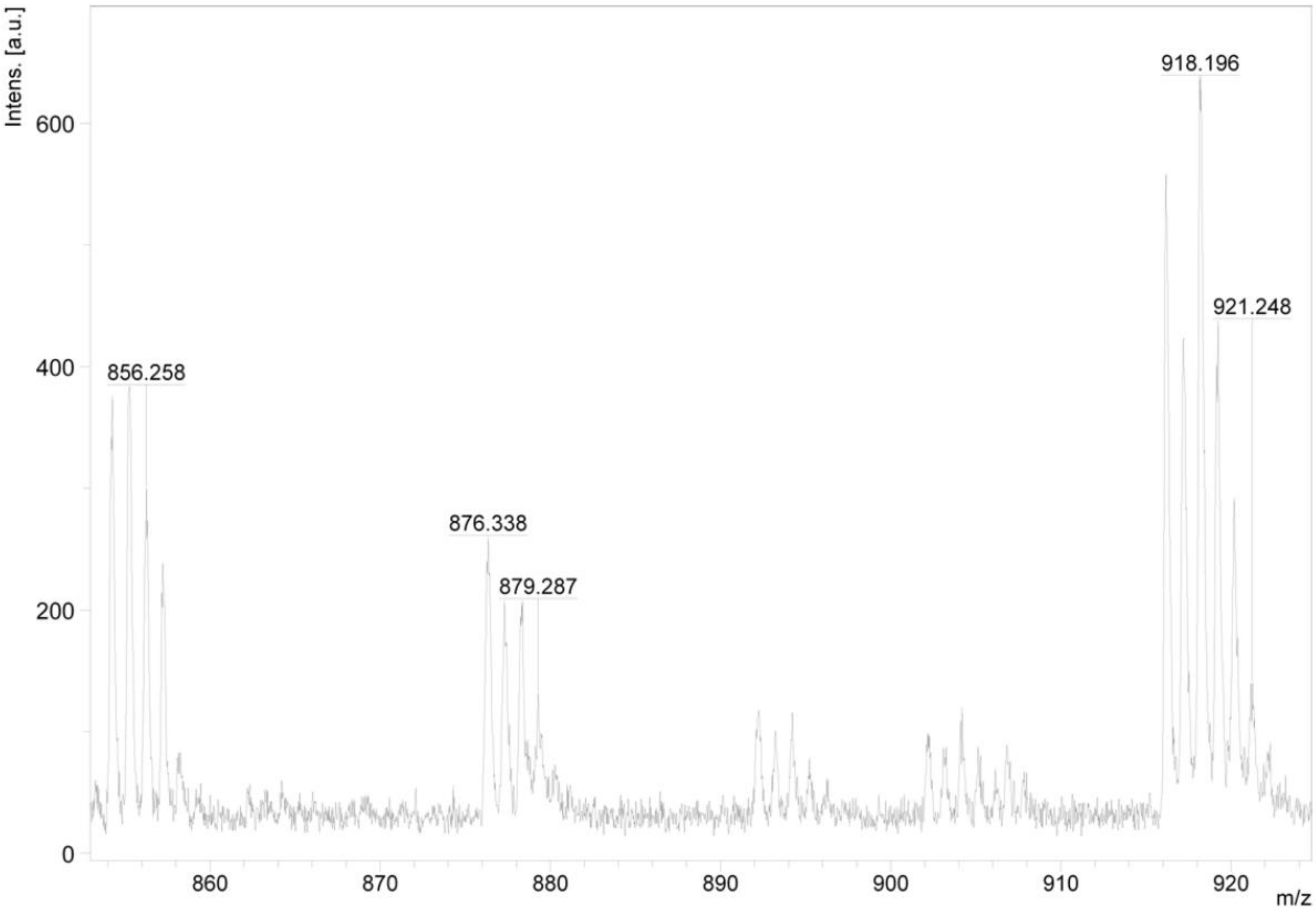

Figura S45. Mass Spectrum of dimethoxy-curcumin.with Cu (II).

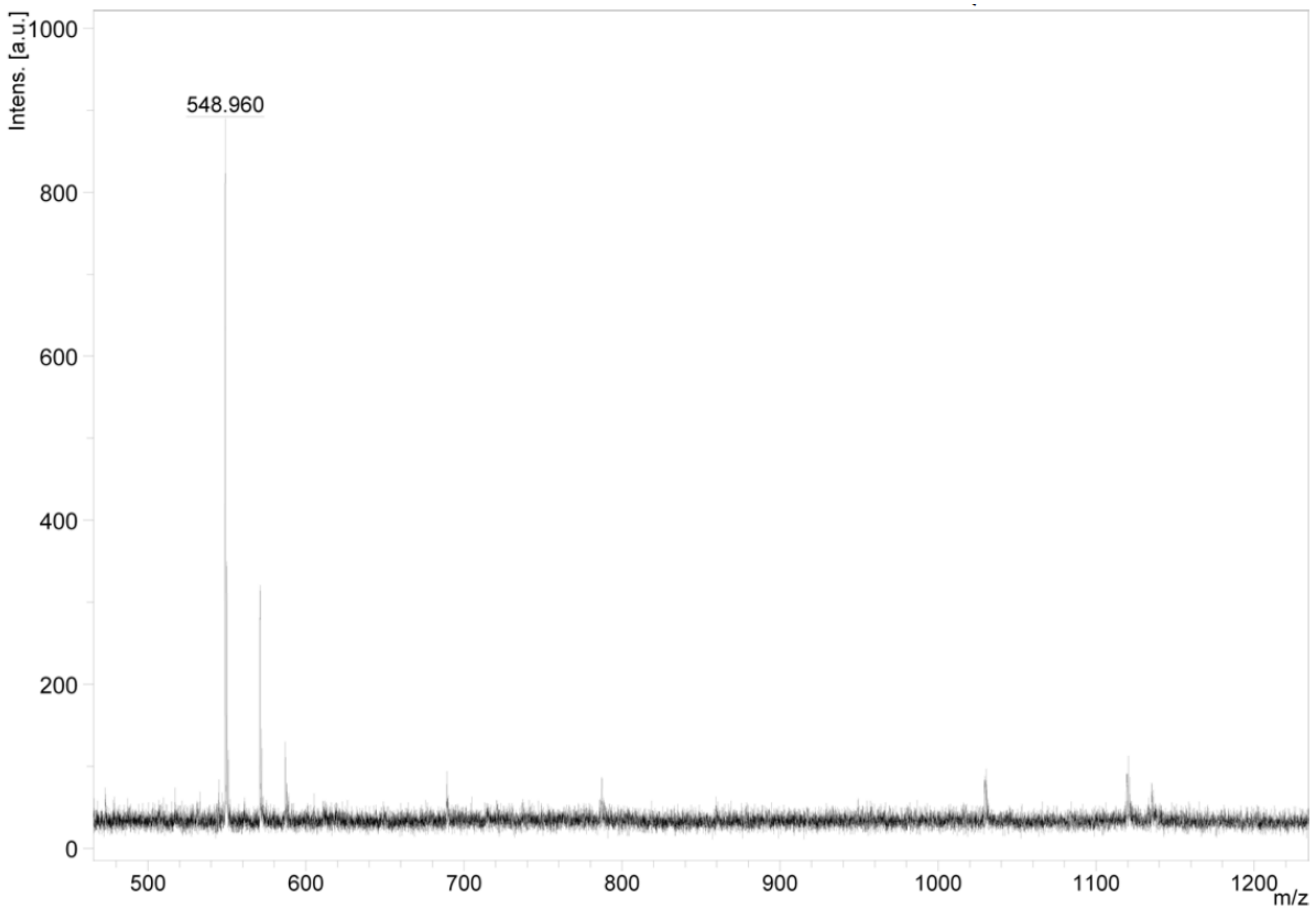

Figura S42. Mass Spectrum of dibenzyl-curcumin.

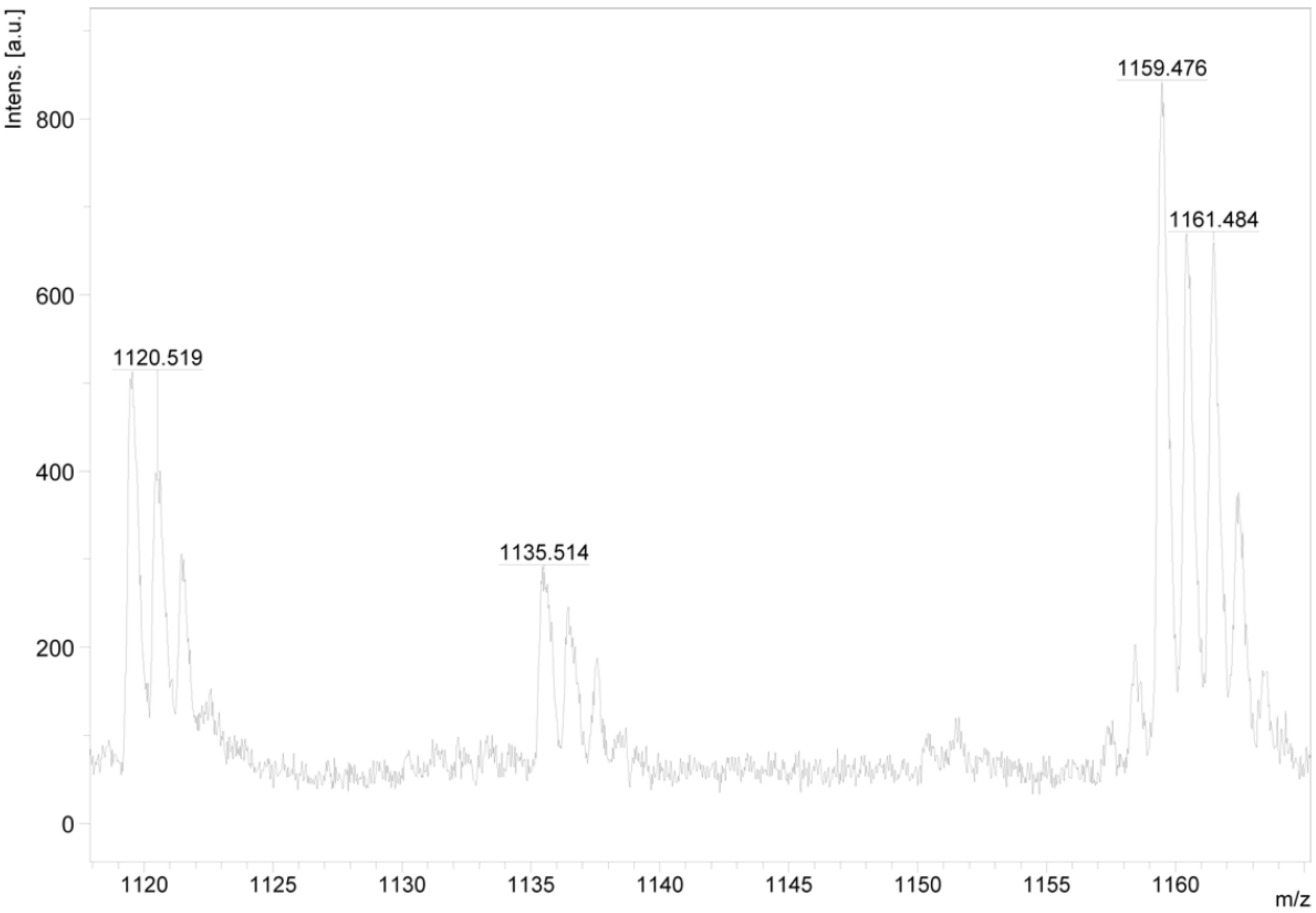

Figura S45. Mass Spectrum of dibenzyl-curcumin.with Cu (II).

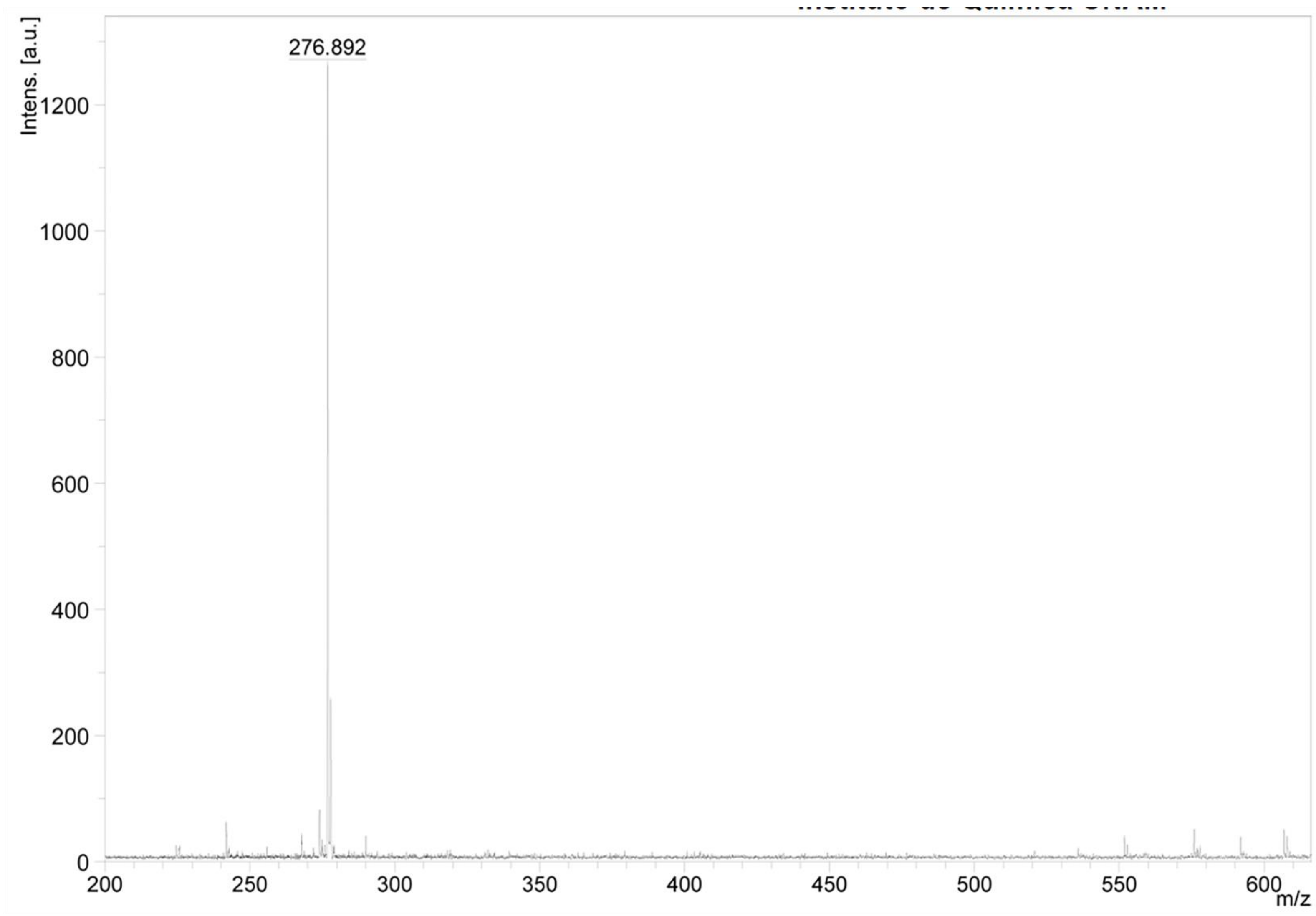

Figura S42. Mass Spectrum of diphenyl-curcumin.

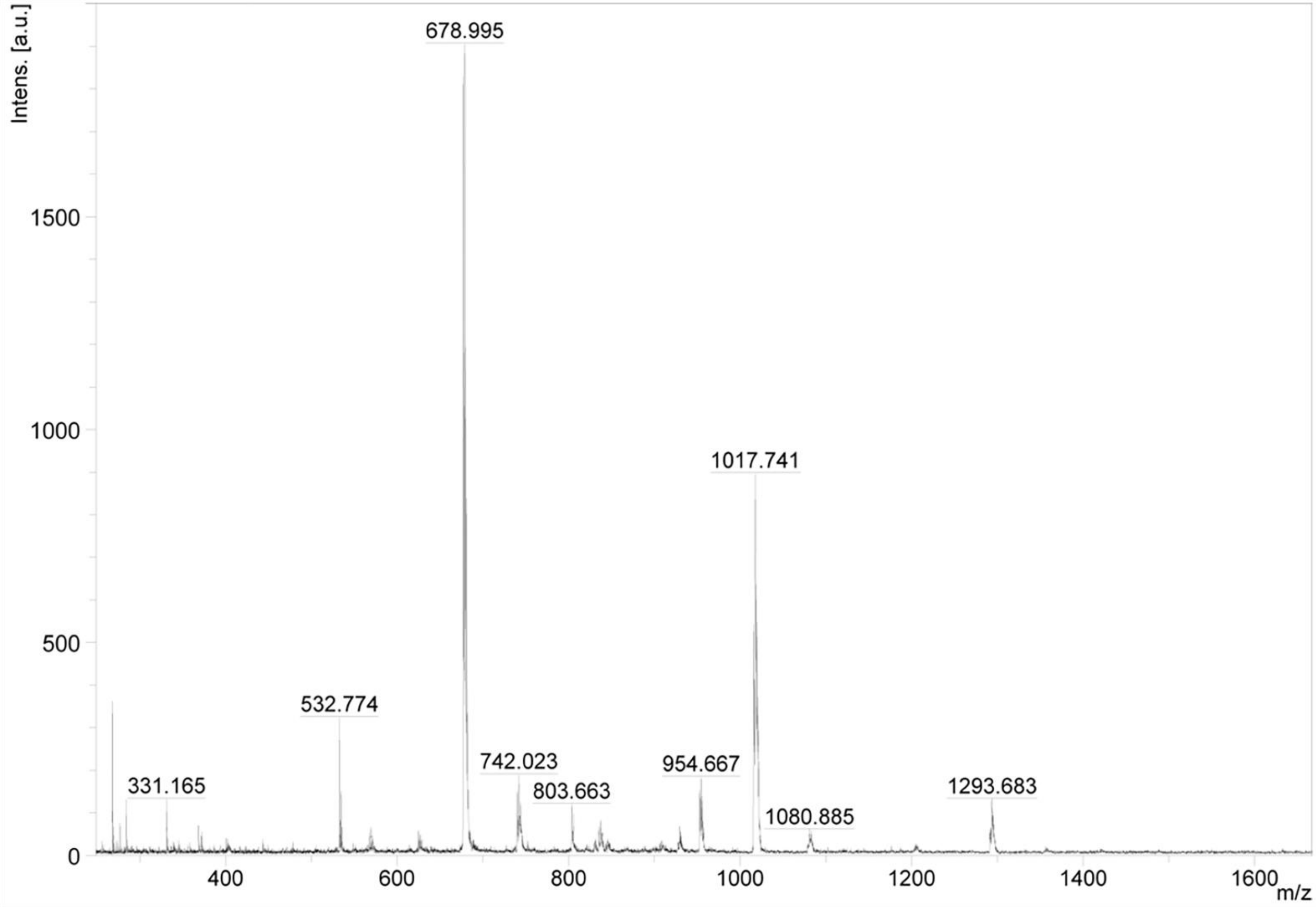

Figura S45. Mass Spectrum of diphenyl-curcumin.with Cu (II).

Table *S1.* Selected Geometric Parameters [Å, °] for Compound_6-10.

Compound_**6**.

Cu(1)-O(2)	1.919(2)
Cu(1)-O(1)	1.933(2)
Cu(1)-O(31)	2.443(6)
O(2)-Cu(1)-O(2)#1	180.0
O(2)-Cu(1)-O(1)	93.58(8)
O(2)#1-Cu(1)-O(1)	86.42(8)
O(1)-Cu(1)-O(1)#1	180.0
O(2)-Cu(1)-O(31)	90.03(18)
O(1)-Cu(1)-O(31)	92.96(15)

Symmetry transformations used to generate equivalent atoms: #1 -x+1,-y,-z+1

Compound_**7**.

Cu(1)-O(1)	1.930(2)
Cu(1)-O(1)#1	1.930(2)
Cu(1)-O(2)	1.937(2)
Cu(1)-O(2)#1	1.937(2)
Cu(1)-O(5)#2	2.590(3)
O(1)-Cu(1)-O(1)#1	180.00(16)
O(1)-Cu(1)-O(2)	92.78(10)
O(1)#1-Cu(1)-O(2)	87.22(10)
O(1)-Cu(1)-O(2)#1	87.22(10)
O(1)#1-Cu(1)-O(2)#1	92.78(10)

O(2)-Cu(1)-O(2)#1	180.00(15)
O(1)-Cu(1)-O(5)#2	88.08(12)
O(1)#1-Cu(1)-O(5)#2	91.92(12)
O(2)-Cu(1)-O(5)#2	87.79(11)
O(2)#1-Cu(1)-O(5)#2	92.21(11)

Compound_**8**.

Cu(1)-O(2)#1	1.891(5)
Cu(1)-O(2)	1.891(5)
Cu(1)-O(1)#1	1.900(4)
Cu(1)-O(1)	1.900(4)
O(2)#1-Cu(1)-O(2)	180.0
O(2)#1-Cu(1)-O(1)#1	92.8(2)
O(2)-Cu(1)-O(1)#1	87.2(2)
O(2)#1-Cu(1)-O(1)	87.2(2)
O(2)-Cu(1)-O(1)	92.8(2)
O(1)#1-Cu(1)-O(1)	180.0

Symmetry transformations used to generate equivalent atoms: #1 -x+2,-y+2,-z+1

Compound_**9a**.

Cu(1)-O(1)#1	1.8954(15)
Cu(1)-O(1)	1.8954(15)
Cu(1)-O(2)	1.9066(15)
Cu(1)-O(2)#1	1.9066(15)
O(1)#1-Cu(1)-O(1)	180.0
O(1)#1-Cu(1)-O(2)	87.69(7)
O(1)-Cu(1)-O(2)	92.31(7)
O(1)#1-Cu(1)-O(2)#1	92.31(7)
O(1)-Cu(1)-O(2)#1	87.69(7)
O(2)-Cu(1)-O(2)#1	180.0

Symmetry transformations used to generate equivalent atoms: #1 -x+1,-y,-z+1

Compound_**9b**.

Cu(1)-O(42A)	1.862(5)
Cu(1)-O(41)	1.9122(14)
Cu(1)-O(1)	1.9144(14)
Cu(1)-O(2)	1.9196(14)
Cu(1)-O(42B)	1.951(4)
Cu(1)-O(42)	1.952(4)
O(42A)-Cu(1)-O(41)	100.3(3)
O(42A)-Cu(1)-O(1)	80.2(3)
O(41)-Cu(1)-O(1)	177.40(7)
O(42A)-Cu(1)-O(2)	168.7(2)
O(41)-Cu(1)-O(2)	85.78(6)

O(1)-Cu(1)-O(2)	93.27(6)
O(41)-Cu(1)-O(42B)	91.6(2)
O(1)-Cu(1)-O(42B)	89.2(2)
O(2)-Cu(1)-O(42B)	175.8(5)
O(41)-Cu(1)-O(42)	92.3(2)
O(1)-Cu(1)-O(42)	88.5(2)
O(2)-Cu(1)-O(42)	176.4(5)

Compound_**10.**

Cu(1)-O(1)#1	1.9030(12)
Cu(1)-O(1)	1.9031(12)
Cu(1)-O(2)	1.9107(13)
Cu(1)-O(2)#1	1.9107(13)
O(1)#1-Cu(1)-O(1)	180.0
O(1)#1-Cu(1)-O(2)	86.89(5)
O(1)-Cu(1)-O(2)	93.11(5)
O(1)#1-Cu(1)-O(2)#1	93.11(5)
O(1)-Cu(1)-O(2)#1	86.89(5)
O(2)-Cu(1)-O(2)#1	180.0

Symmetry transformations used to generate equivalent atoms: #1 -x+1,-y,-z+1

Table S2. Selected Torsion Angles for Compounds **6** to **10**

Torsion Angle	Compound **6**	Compound **7**	Compound **8**	Compound **9a**	Compound **9b**	Compound **10**
C(8)-C(1)-C(2)-C(3)	176.0(3)	-168.7(4)	179.9(7)	172.7(2)	-174.2(2)	177.96(18)
C(1)-C(2)-C(3)-C(4)	178.1(3)	-134.9(4)	178.2(8)	-176.7(2)	-168.0(2)	178.8(2)
C(2)-C(3)-C(4)-C(5)	179.5(3)	-179.7(4)	179.6(8)	177.7(2)	-173.2(2)	178.4(2)
C(3)-C(4)-C(5)-C(6)	175.5(3)	-172.6(4)	-178.1(8)	176.5(2)	174.7(2)	177.6(2)
C(4)-C(5)-C(6)-C(7)	175.5(3)	-148.0(5)	178.1(8)	-173.3(2)	-169.7(2)	-175.8(2)
C(5)-C(6)-C(7)-C(14)	177.5(3)	-161.5(4)	-177.8(8)	173.5(3)	-179.2(4)	179.62(18)
C(48)-C(41)-C(42)-C(43)					-176.4(2)	
C(41)-C(42)-C(43)-C(44)					-168.3(5)	
C(42)-C(43)-C(44)-C(45)					-173.2(10)	
C(43)-C(44)-C(45)-C(46)					173.8(10)	
C(44)-C(45)-C(46)-C(47)					-174.6(16)	
C(45)-C(46)-C(47)-C(54)					179.6(16)	
